# Glycolysis-mTORC1 crosstalk drives proliferation of patient-derived endometrial cancer spheroid cells with ALDH activity

**DOI:** 10.1038/s41420-024-02204-y

**Published:** 2024-10-11

**Authors:** Haruka Ueda, Tatsuya Ishiguro, Yutaro Mori, Kaoru Yamawaki, Koji Okamoto, Takayuki Enomoto, Kosuke Yoshihara

**Affiliations:** 1 Department of Obstetrics and Gynecology, Niigata University Graduate School of Medical and Dental Sciences, Niigata, Japan; 2 Advanced Comprehensive Research Organization, Teikyo University, Tokyo, Japan

**Keywords:** Endometrial cancer, Cancer stem cells

## Abstract

Cancer stem cells are associated with aggressive phenotypes of malignant tumors. A prominent feature of uterine endometrial cancer is the activation of the PI3K–Akt–mTOR pathway. In this study, we present variations in sensitivities to a PI3K–Akt–mTORC1 inhibitor among in vitro endometrial cancer stem cell-enriched spheroid cells from clinical specimens. The in vitro sensitivity was consistent with the effects observed in in vivo spheroid-derived xenograft tumor models. Our findings revealed a complementary suppressive effect on endometrial cancer spheroid cell growth with the combined use of aldehyde dehydrogenase (ALDH) and PI3K–Akt inhibitors. In the PI3K–Akt–mTORC1 signaling cascade, the influence of ALDH on mTORC1 was partially channeled through retinoic acid-induced lactate dehydrogenase A (LDHA) activation. LDHA inhibition was found to reduce endometrial cancer cell growth, aligning with the effects of mTORC1 inhibition. Building upon our previous findings highlighting ALDH-driven glycolysis through GLUT1 in uterine endometrial cancer spheroid cells, curbing mTORC1 enhanced glucose transport via GLUT1 activation. Notably, elevated LDHA expression correlated with adverse clinical survival and escalated tumor grade, especially in advanced stages. Collectively, our findings emphasize the pivotal role of ALDH–LDHA–mTORC1 cascade in the proliferation of endometrial cancer. Targeting the interaction between mTORC1 and ALDH-influenced glycolysis holds promise for developing novel strategies to combat this aggressive cancer.

## Introduction

Uterine endometrial cancer is a major gynecological ailment. Although early-stage and low-grade uterine endometrial cancers present relatively mild behaviors, standard chemotherapy regimens fail to adequately address high-grade or metastatic tumors [1]. A common molecular characteristic of both type I and type II endometrial cancers is the dysregulation of the PI3K–Akt–mTORC1 signaling pathway [2]. Positioned as a downstream target, mTORC1 is influenced by numerous oncogenic pathways in cancer, such as the PI3K–Akt and MAPK pathways [3]. Furthermore, the mTORC1 complex is modulated by growth factors, amino acids, energy levels, and stress, and it plays a role in cell growth through protein, lipid, and nucleotide synthesis as well as autophagy [3]. Similar to many cancers, most endometrial cancers exhibit genomic aberrations in the PI3K–Akt–mTORC1 signaling pathway, which often lead to mTORC1 hyperactivation [4]. Thus, therapies targeting the PI3K–Akt–mTORC1 signaling could potentially enhance patient outcomes in endometrial cancer [3, 5]. However, results from a recent phase I/II trial involving an mTOR inhibitor combined with an aromatase inhibitor, anastrozole, showed limited improvements in hormone receptor-positive recurrent or metastatic endometrial cancer patients, with an overall response rate of 24.5% [6]. Another phase II clinical trial demonstrated that dual PI3K/mTOR inhibitors offered modest clinical benefits (overall response rate: 16%; duration of response: 4.2 months) with manageable side effects [7]. Further investigation into the mechanism of mTORC1 signaling and formulating methods for identifying patients who could benefit are crucial priorities.

Cancer stem cells, identified as primary contributors to cancer origination, when eliminated, significantly retard cancer progression [8]. Aldehyde dehydrogenase (ALDH) serves as a specific marker for many types of cancer stem cells [9]. ALDH, pivotal in retinoid metabolism and toxic aldehyde removal, influences cancer progression, stemness, and chemotherapy resistance across various cancers. Notably, among the 19 ALDH isoforms, ALDH1A1, ALDH1A3, and ALDH3A1 are tied to cancer stem cells [10]. We previously identified ALDH as a functional marker for both ovarian and uterine endometrial cancer stem cells; specifically, both endometrial cancer spheroid cells exhibiting high ALDH activity and exogenous ALDH1A1 overexpressing cells have demonstrated substantial tumorigenesis potential in vivo [11, 12]. In particular, we identified ALDH1A1 as the specific isoform marking uterine endometrial cancer stem cells and revealed the role of the ALDH–GLUT cascade in paclitaxel resistance in these cells [12]. However, the ALDH mechanism in endometrial cancer proliferation warrants further investigation.

Patient-derived tridimensional cells, encompassing spheroid and organoid cells from clinical specimens, retain several clinical traits. They are invaluable platforms for assessing drug sensitivity in numerous solid cancers, including ovarian and uterine endometrial cancers [13, 14]. Our previous research indicated that gynecological cancer patient-derived spheroid cells exhibit cellular diversity and potent tumorigenic capabilities in vivo, which are key features typically associated with cancer stem cells [11, 12, 15]. Moreover, our spheroid cells have demonstrated efficacy in drug sensitivity determination [16], aligning with the well-established role of organoid cells in drug sensitivity assays [17].

In this study, we aimed to investigate the sensitivity of inhibitors targeting the PI3K–Akt–mTORC1 signaling, employing uterine endometrial cancer patient-derived spheroid cells. The findings of this study underscore that targeting ALDH–LDHA–mTORC1 signaling presents a novel treatment strategy for aggressive uterine endometrial cancer.

## Results

### PI3K–Akt–mTOR signaling patterns in human uterine endometrial cancer spheroid cells

**Fig. 1:**
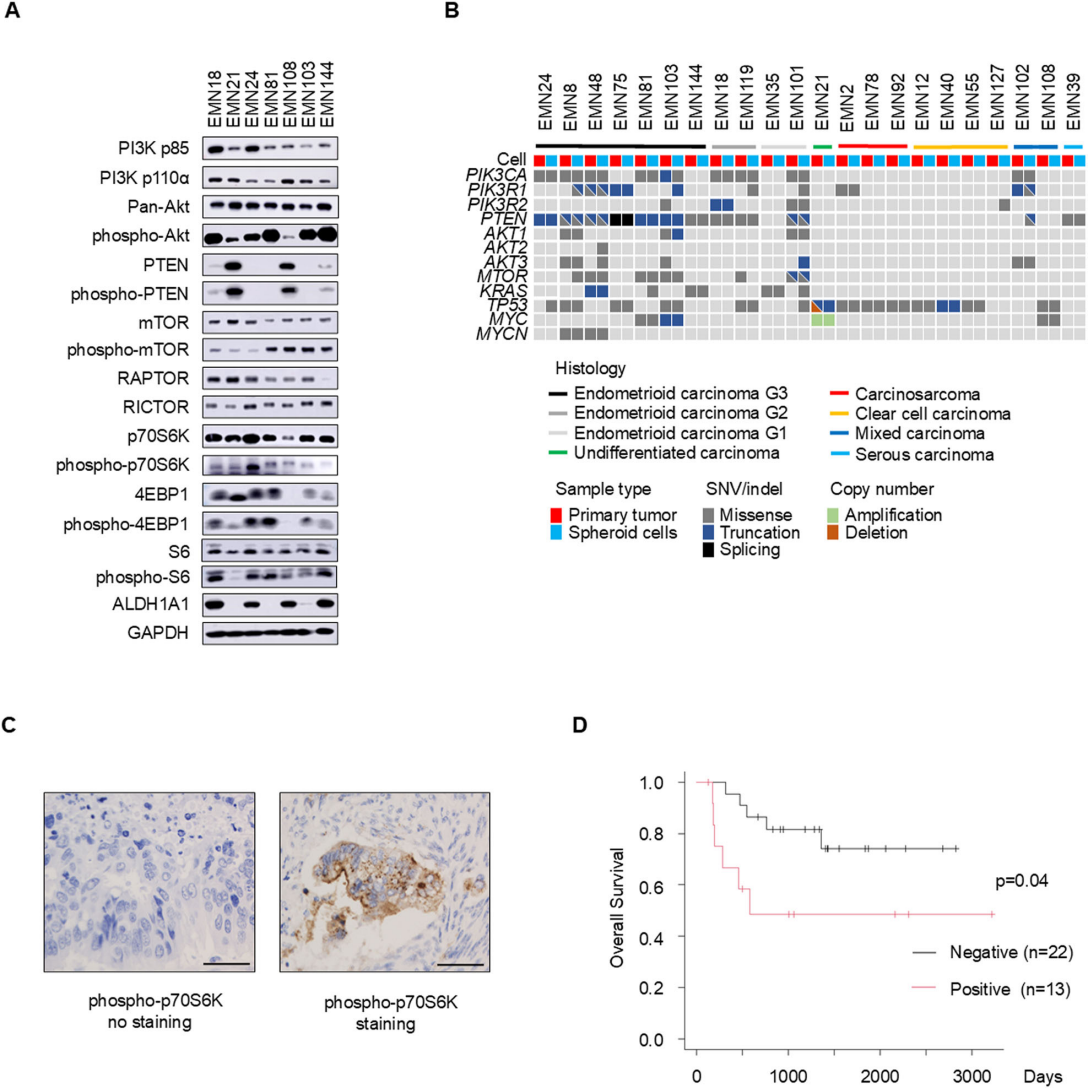
PI3K–Akt–mTOR signaling expression in endometrial cancer. See also Supplementary Figs. S1, S2 and Supplementary Table S1. **A** Western blot of endometrial cancer spheroid cells. **B** Targeted sequencing analysis of primary tumors and spheroid cells. **C** Representative immunostaining images for phospho-p70S6K-negative (left) and phospho-p70S6K-positive (right). Scale bars: 50 µm. **D** Kaplan–Meier plots of overall survival in patients with advanced-stage endometrial cancer stratified by phospho-p70S6K positivity (red, *n* = 13) and negativity (black, *n* = 22) (*p* = 0.04). Notably, most patients received first-line taxane and platinum-based chemotherapy.

First, we evaluated the protein expression and gene mutation profile of PI3K–Akt–mTOR signaling to delineate the signaling status in endometrial cancer spheroid cells. Western blot analysis of seven distinct endometrial cancer spheroid cells revealed variations in the expression levels of signaling factors, including phospho-Akt, phospho-PTEN, and phospho-p70S6K. Their expression did not correlate with the ALDH1A1 expression within the spheroid cell types (Fig. 1A).

Subsequently, an immunohistochemical evaluation of spheroid-derived xenograft tumors was undertaken alongside the original clinical cancer tumor specimens. This highlighted that the expression patterns of phospho-Akt, phospho-p70S6K, and phospho-PTEN echoed the results observed in the aforementioned western blot analysis of spheroid cells. Notably, although a heterogeneous expression was observed across tumors, numerous cancer cells exhibited increased phospho-Akt expression in tumors originating from or derivatives of EMN24 and EMN144 cells. Furthermore, the tumors from EMN24 cells displayed pronounced phospho-p70S6K expression, and the tumors from EMN108 cells showed evident phospho-PTEN expression. An immunohistochemical evaluation of spheroid cells, including EMN24, EMN108, and EMN144, showed a similar expression of phosphorylated (phospho-) Akt, PTEN, p70S6K, mTOR, 4EBP1, and S6 with the original tumors (Supplementary Fig. S1A). In a broader context, mutation profiles across 22 endometrial cancer spheroid cells closely resembled those witnessed in the original clinical tumor specimens (Fig. 1B) [12].

Among these PI3K–Akt–mTORC1-related signaling factors, a previous study showed that phospho-p70S6K is a predictive tool for the outcomes of patients with type II endometrial cancer when used as an immunohistochemistry (IHC)-based marker [2]. This was particularly significant among markers related to the activation of the PI3K–Akt–mTORC1 signaling pathway [2]. Furthermore, the phosphorylation observed at T389 showcased a connection with malignancy-related p70S6K activity [18]. Merging this insight with the observed expression results of phospho-p70S6K in both spheroid cells and spheroid-derived xenograft tumors, we sought to confirm the expression of phospho-p70S6K in 35 clinically advanced endometrial cancer tissue specimens through immunohistochemical staining (Fig. 1C). Kaplan–Meier survival analyses showed that an elevated phospho-p70S6K expression may be correlated with overall survival (Fig. 1D, *p* = 0.04), not with progression-free survival (Supplementary Fig. S2A). The expression was not correlated with histological grade or clinical stage in these advanced-stage cases (Supplementary Fig. S2B, C, Fisher’s exact test). Although the conclusions drawn from a limited case pool remain preliminary, our findings suggest that phospho-p70S6K expression may be linked to a poor prognosis in advanced-stage uterine endometrial cancer.

### PI3K Inhibitors curbing the proliferation of endometrial cancer spheroid cells

With the aforementioned results of phospho-p70S6K expression being related to the activation of the PI3K–Akt–mTORC1 signaling pathway [2], we then explored the effects of PI3K–Akt–mTORC1 signaling in endometrial cancer spheroid cells. Our in vitro cancer spheroid model displayed superior efficacy compared to the cancer stem cell model [11, 12] and existing in vitro drug sensitivity evaluations [16]. Riding on this fundamental advantage, we first assessed the sensitivity of endometrial cancer cells toward PI3K inhibitors. The particular focus was on Alpelisib, a prominent PI3K inhibitor deployed in clinical trials to treat breast and several other cancers [19, 20].

**Fig. 2:**
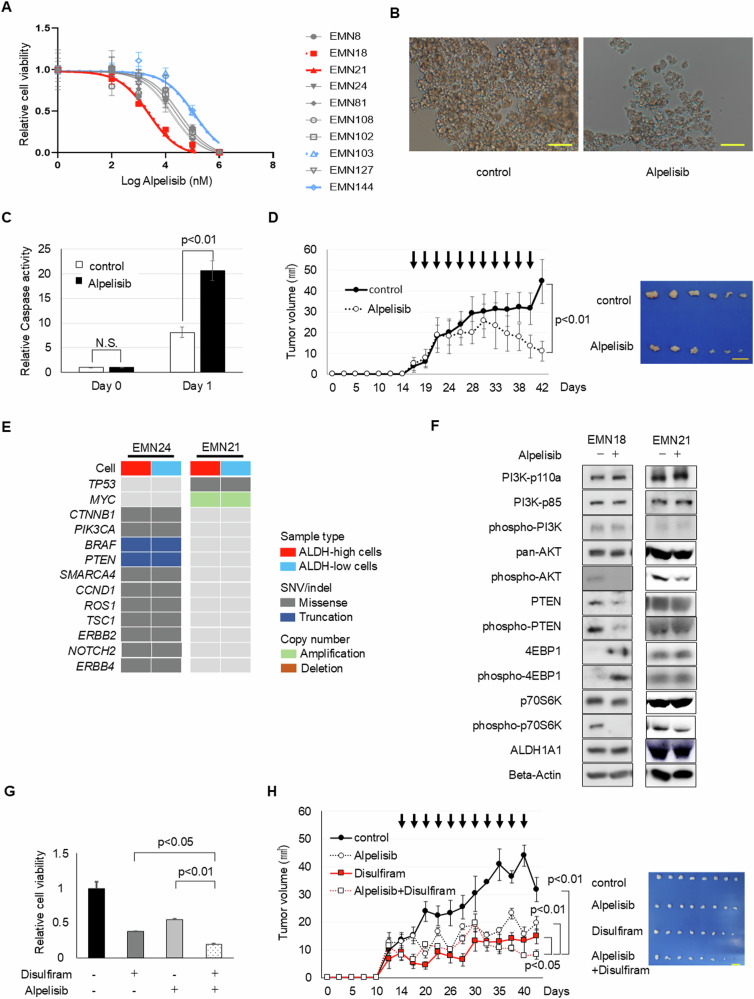
Combination therapy of Alpelisib and ALDH inhibitor inhibits endometrial cancer spheroid cell progression. See also Supplementary Figs. S3 and S4. **A** Spheroid cell responses to varied Alpelisib concentrations over 4 days. **B** Bright-phase images of EMN18 spheroids after 4 days of 5 nM Alpelisib treatment. Scale bars: 100 µm. **C** Caspase activity in EMN18 cells pre- and post-Alpelisib exposure. **D** Xenograft tumor volumes (mean ± SEM) post-subcutaneous injection of 5 × 10^5^ EMN18 spheroids. Mice in the Alpelisib group were intraperitoneally administered Alpelisib (15 mg/kg), whereas the control group received DMSO. Data from *n* = 6 independent experiments, *p* < 0.01, Student’s *t*-test. Scale bars: 10 mm. **E** Target sequencing of ALDH-high and ALDH-low spheroid cells. Spheroid cells were separated under ALDH activity after ALDEFLUOR staining (StemCell Technologies Inc.) using a FACS Aria III Cell Sorter (BD Biosciences). **F** Western blot of spheroid cells treated with or without Alpelisib. **G** Relative EMN21 spheroid cell viability under specified treatments over 4 days. **H** Xenograft tumor volumes (mean ± SEM) from 1 × 10^6^ EMN21 spheroids, treated in vivo with combinations of Alpelisib (15 mg/kg) and/or disulfiram (40 mg/kg). *n* = 8, Student’s *t*-test. Xenograft tumor images post-excision on day 42 are on the right. Scale bar: 10 mm.

Upon assessment, we categorized the endometrial cancer cells into three distinct sensitivity groups with respect to Alpelisib:

1. High-sensitivity group: EMN18 and EMN21 cells.
2. Low-sensitivity group: EMN103 and EMN144 cells.
3. Intermediate-sensitivity group: Comprising the remaining cell types (Fig. 2A, B).

Our examination of other PI3K inhibitors, namely Taselisib and Copanlisib, showed sensitivity patterns similar to those observed for Alpelisib (Supplementary Fig. S3A, B). Cancer cases that harbored the activating mutation *PIK3CA* H1047R in the coding exon 20 (a kinase-activating mutation) reliably respond to Alpelisib treatment [21, 22]. This mutation is one of the most common *PIK3CA* mutations in gynecologic cancers [23]. In our spheroids, EMN18 cells harboring this *PIK3CA* mutation are one of the sensitive cells to Alpelisib.

Alpelisib treatment enhanced caspase levels when the spheroid cellular growth was inhibited (Fig. 2B, C). To compare in vivo sensitivity against its in vitro counterpart, we administered Alpelisib to NOG mice thrice weekly. The outcomes were illuminating: the xenograft tumors stemming from EMN18 and EMN21 spheroid cells (from the in vitro high-sensitivity group) exhibited a decline post-Alpelisib treatment (Fig. 2D). Moreover, tumors from EMN81 spheroid cells (intermediate-sensitive) also exhibited growth suppression (Supplementary Fig. S3C). Nonetheless, tumors originating from EMN144 cells (low-sensitivity group) remained unaffected by Alpelisib treatment (Supplementary Fig. S3D). Collectively, these findings highlight that the Alpelisib sensitivity observed in our in vitro spheroid model is consistent with the findings of our in vivo applications.

### Additive effects of PI3K and ALDH inhibitors on endometrial cancer spheroid cells’ growth

Next, we elucidated the interactions between PI3K–Akt–mTORC1 signaling and cancer stemness using ALDH, which was found to be a functional marker of uterine endometrial cancer stem cells in our previous study [12]. A targeted sequencing analysis revealed identical mutational profiles between ALDH-high and ALDH-low spheroid cells (Fig. 2E). Moreover, despite the suppression of phospho-Akt and phospho-p70S6K expression post-Alpelisib treatment in sensitive spheroid cells (Fig. 2F), we observed no discernable differences in the in vitro sensitivity to Alpelisib between ALDH-high and ALDH-low cells (data not shown). This observation was consistent with the changes in ALDH activity or ALDH1A1 expression (Fig. 2F and Supplementary Fig. S4A). Further, Alpelisib treatment diminished phospho-p70S6K expression in both ALDH-high and ALDH-low cells (Supplementary Fig. S4B). Given the above, we postulated that ALDH activity might be independent of PI3K sensitivity. To investigate this hypothesis, we co-treated spheroid cells with disulfiram (an ALDH inhibitor) and Alpelisib. This combination treatment led to a remarkable inhibition of spheroid growth (Fig. 2G).

Validating these in vitro findings, we introduced this co-treatment to spheroid cell-transplanted mice. Tumors post-co-treatment were roughly half the size compared with those treated solely with Alpelisib. This was consistent for both high-sensitivity (EMN21, Fig. 2H) and intermediate-sensitivity (EMN24, Supplementary Fig. S4C) cell groups. Additionally, consistent with in vitro results, Alpelisib alone did not alter the ALDH activity in xenograft tumors (Supplementary Fig. S4D). Thus, although ALDH activity alone does not directly affect PI3K inhibition, a combination of ALDH and PI3K inhibitors results in an additive inhibition of endometrial cancer progression.

### Additive inhibition of endometrial cancer spheroid cells by combining Akt and ALDH inhibitors

**Fig. 3:**
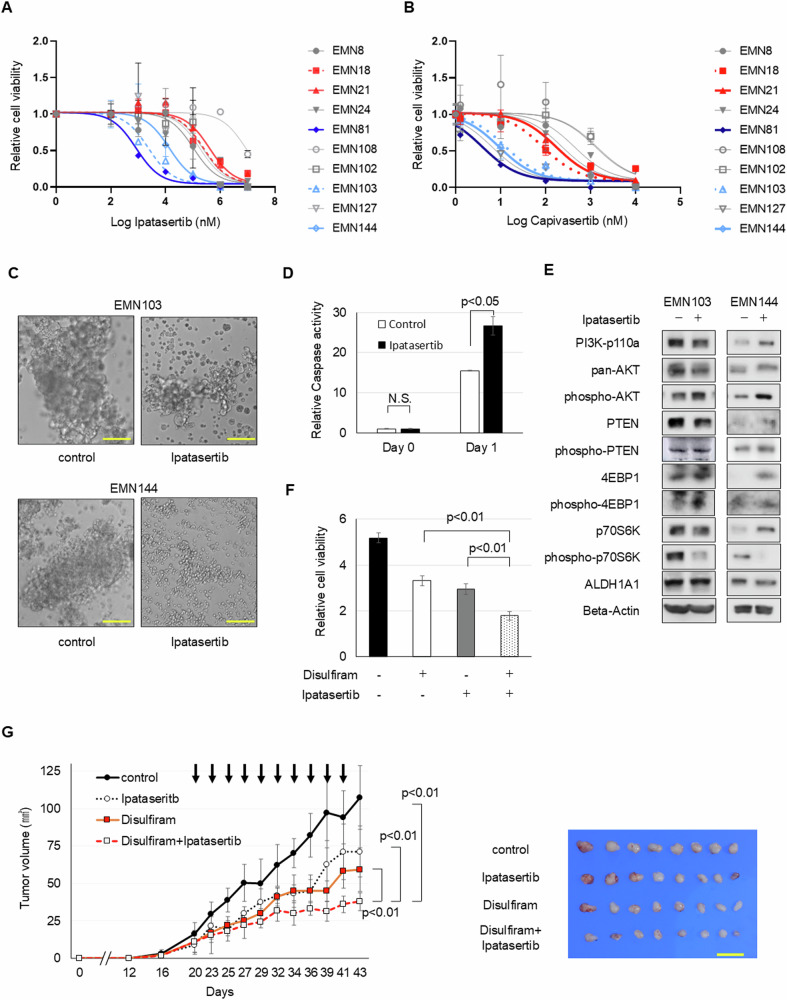
Ipatasertib and ALDH inhibitor combination impacts endometrial cancer cell progression. See also Supplementary Fig. S5. Responses of spheroid cells to different concentrations of (**A**) Ipatasertib and (**B**) Capivasertib after incubation for 4 days. **C** Bright-phase images of EMN103 and EMN144 spheroid cells (4 days after Ipatasertib treatment). Scale bars, 100 µm. **D** Caspase activity before and after 1 nM Ipatasertib treatment to EMN144 cells. **E** Western blot analyses of spheroid cells in the presence or absence of Ipatasertib treatment. **F** Relative cell viability in the presence or absence of 100 nM Ipatasertib and 0.5 µM disulfiram treatment for 4 days. **G** Volumes (mean ± SEM) of xenograft tumors from 1 × 10^6^ EMN144 spheroid cells with the presence or absence of Ipatasertib (15 mg/kg) and/or disulfiram (20 mg/kg) after in vivo treatment. *n* = 8, Student’s *t*-tests. Xenograft tumor images post-excision on day 43 are on the right. Scale bar: 10 mm.

We shifted our focus from PI3K inhibitors to studying the effects of Akt inhibitors on endometrial cancer spheroid cells. Notably, cells that exhibited low-to-intermediate sensitivity to PI3K inhibitors—specifically, the EMN81, EMN103, and EMN144 cell lines (Fig. 2A and Supplementary Fig. S3A,B)—responded to low-dose treatment with Akt inhibitors, Ipatasertib and Caplivasertib (Fig. 3A–C). This sensitivity was marked by an uptick in activated caspases post-Ipatasertib treatment, implying cytotoxic effects (Fig. 3D).

However, although EMN24 cell-derived xenograft tumors demonstrated resistance to Ipatasertib in vitro, they remained unresponsive to the drug even in vivo (Supplementary Fig. S5). These findings highlight the potential of baseline Akt activity, particularly linked to PTEN mutations, as a valuable predictor for Akt inhibitor sensitivity (Fig. 1A, B) [24].

Although phospho-p70S6K expression decreased after treatment with the Akt inhibitor, ALDH activity or expression remained unchanged (Fig. 3E). These observations led us to hypothesize that ALDH activity remained unaffected by Akt inhibition. However, dual inhibition of both Akt and ALDH might collectively hinder endometrial cancer cell growth. To test this, we investigated the combined impact of ALDH and Akt inhibitors. Combination treatment hindered cancer cell proliferation both in vitro and in vivo (Fig. 3F–G). Consistent with our findings on PI3K inhibitors, this co-treatment approach further validated the additive repression of endometrial cancer progression, independent of any influence of ALDH activity on Akt inhibition.

### mTOR inhibitor reduces the proliferation of endometrial cancer spheroid cells with ALDH activity

**Fig. 4:**
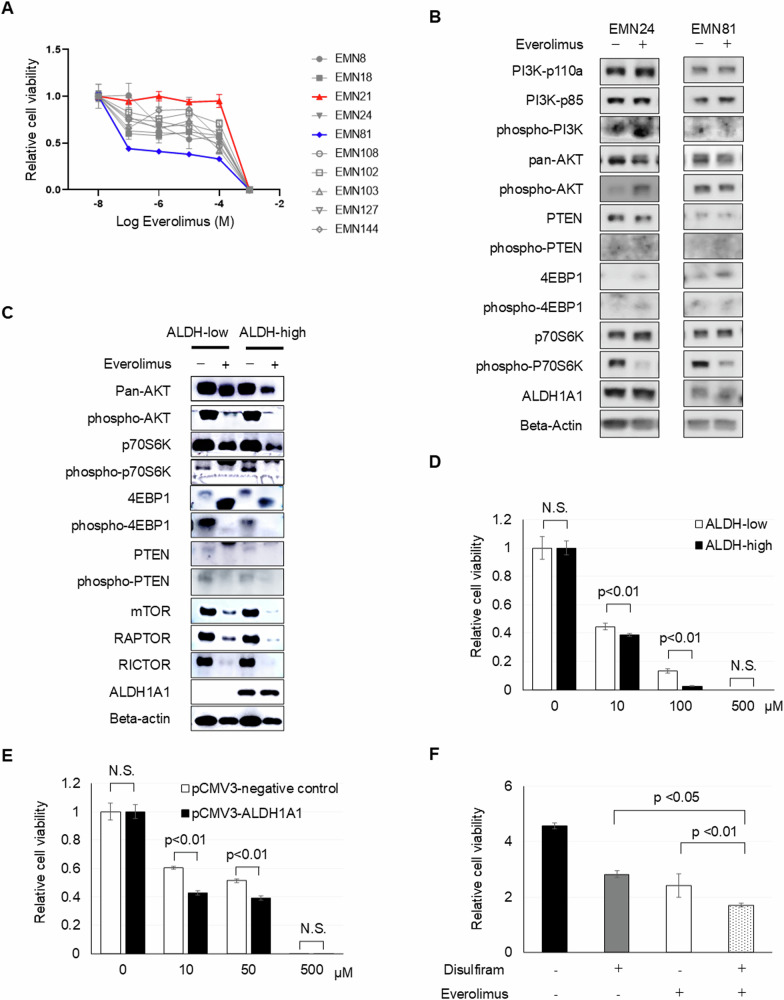
mTOR inhibitor blocks the proliferation of endometrial cancer spheroid cells. See also Supplementary Figs. S6 and S7. **A** Responses of spheroid cells to different concentrations of everolimus after incubation for 4 days. **B** Western blot analyses of spheroid cells in the presence or absence of everolimus treatment. **C** Western blot analyses of ALDH-low and ALDH-high spheroid cells in the presence or absence of everolimus treatment. **D** Relative ALDH-high and ALDH-low spheroid cell viability with the indicated everolimus in vitro treatment for 4 days. **E** Relative infected spheroid cell viability with the indicated everolimus in vitro treatment for 4 days. **F** Relative cell viability in the presence or absence of 5 µM everolimus and 10 µM disulfiram treatment for 4 days.

Our experiments with PI3K and Akt inhibitors indicated that PI3K/Akt activity was independent of ALDH activity. Our investigation subsequently focused on understanding the influence of mTOR inhibition on endometrial cancer spheroid cell proliferation. In vitro sensitivity assays revealed varying responses to mTOR inhibitors, everolimus, and Torin1 (Fig. 4A and Supplementary Fig. S6A). Notably, the range of sensitivities was more consistent for the inhibitors tested, unlike PI3K and Akt inhibitors (Figs. 2A, 3A, B and Supplementary Fig. S3A, B). Following mTOR inhibitor administration, there was a decline in the levels of both phospho-p70S6K and phospho-4EBP1 across the spheroid cell population (Fig. 4B). This decrease in phospho-p70S6K levels was greater in ALDH-high cells than in ALDH-low cells (Fig. 4C and Supplementary Fig. S6B). Moreover, the ALDH-high cells exhibited increased sensitivity to everolimus compared to their ALDH-low counterparts (Fig. 4D). It was discerned that the exogenous overexpression of ALDH1A1 further augmented this sensitivity to everolimus (Fig. 4E and Supplementary Fig. S7). Collectively, these findings support the idea that cells with higher ALDH activity inherently possess increased mTOR activation compared to cells with low ALDH activity. Alternatively, combination treatment with disulfiram and everolimus also hindered the spheroid cell proliferation (Fig. 4F). Although ALDH activity appears to be linked to mTORC1 activity, a combination of ALDH and mTORC1 inhibitors additively impedes endometrial cancer progression.

### Endometrial cancer spheroid cells exhibiting high ALDH activity show enhanced sensitivity to mTOR inhibitors

**Fig. 5:**
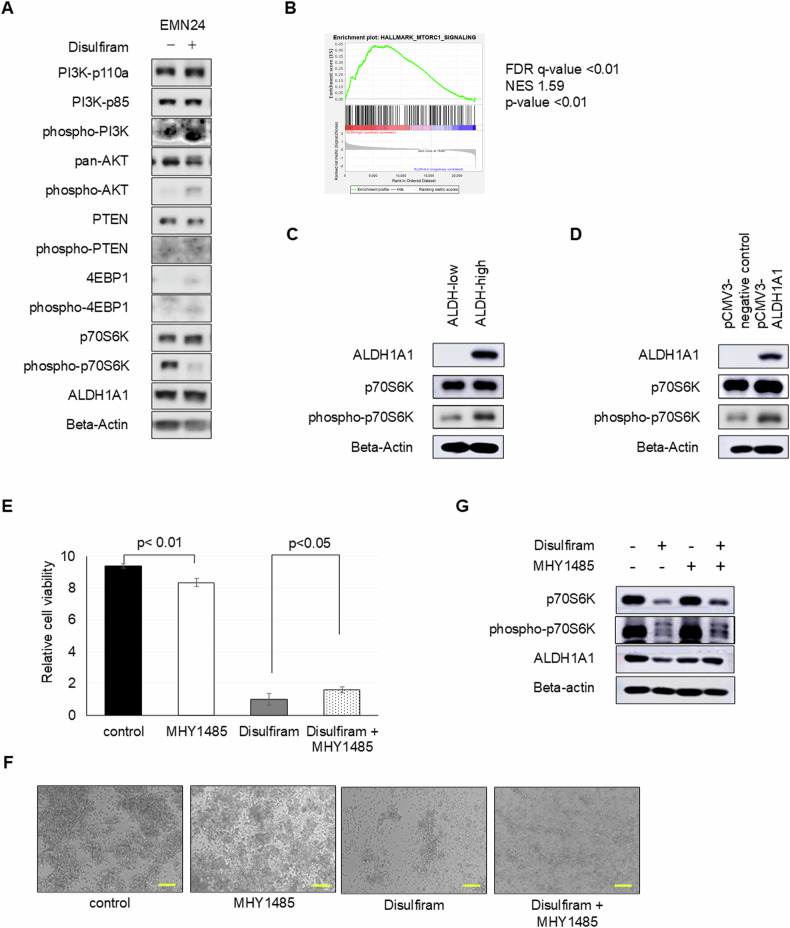
ALDH inhibitor blocks the proliferation of endometrial cancer cells via mTOR activity (EMN24 cells). See also Supplementary Fig. S8. **A** Western blot analyses of spheroids cells after disulfiram treatment for 24 h. **B** Gene set enrichment analyses of gene expression profiles between ALDH-high and ALDH-low cells (HALLMARK_MTORC1_SIGNALING, genes upregulated through activation of mTORC1 complex). **C** Western blot analyses of ALDH-high and ALDH-low spheroids cells. **D** Western blot analyses of the infected spheroids cells. **E** Relative spheroid cell viability in the presence or absence of 10 µM disulfiram and/or 5 µM MHY1485 in vitro treatment for 4 days. **F** Bright-phase images of spheroid cells in the presence or absence of disulfiram and/or MHY1485 in vitro treatment for 4 days. Scale bars: 100 µm. **G** Western blot analyses of spheroids cells after disulfiram and/or MHY1485 treatment for 24 h.

Based on the aforementioned results, we speculated that increased ALDH activity might partially enhance mTOR sensitivity in endometrial cancer. To reveal the relationship between ALDH and mTOR, we examined changes in the expression of the PI3K–Akt–mTOR signaling pathway after manipulating ALDH activity. Disulfiram decreased phospho-p70S6K levels without affecting phospho-PI3K and phospho-Akt levels (Fig. 5A and Supplementary Fig. S8A). Additionally, gene set enrichment analysis (GSEA) revealed the ALDH-high cells preferentially expressed genes included in the gene set of hallmark of mTORC1 signaling (false discovery rate [FDR] *q*-value < 0.01, normalized enrichment score [NES] 1.59, *p*-value < 0.01) [22], genes upregulated after ectopically expressing eIF4E, and genes upregulated in control cells compared with eIF4GI-silenced cells [23, 24] (Fig. 5B and Supplementary Fig. S8B). Moreover, ALDH-high cells clearly expressed more phospho-p70S6K than ALDH-low cells (Fig. 5C). The exogenous overexpression of ALDH1A1 that led to ALDH activation [12] ultimately increased phospho-p70S6K levels (Fig. 5D and Supplementary Fig. S7B). These results suggested that ALDH activity primarily affected mTORC1 activation rather than PI3K or Akt. To further confirm the relationship between ALDH and mTORC1 signaling, we determined whether mTOR activation could rescue the inhibitory effect of ALDH inhibitor on cancer cells. As expected, mTOR activator MHY1485 partially mitigated disulfiram-induced cytotoxicity in endometrial cancer spheroid cells (Fig. 5E, F and Supplementary Fig. S8C–F). Moreover, western blot analysis indicated that MHY1485 could partially revert the disulfiram-mediated reduction in phospho-p70S6K expression (Fig. 5G and Supplementary Fig. S8G).

### LDHA bridges ALDH activity and mTORC1 activation in endometrial cancer spheroid cells

To understand ALDH’s impact on mTOR signaling in endometrial cancer cells, we characterized its functional isoforms. Notably, of the 19 ALDH isoforms with analogous catalytic functions, ALDH1A1 predominantly dictates its activity [25]. Our findings confirmed that endometrial cancer spheroid cells predominantly expressed ALDH1A1 over other ALDH isoforms [12]. A pivotal function of ALDH1A1 includes the conversion of retinol to retinoic acids, driving cancer proliferation through multifaceted mechanisms [26, 27]. Corroborating our premise that ALDH-mediated retinoic acid modulates endometrial cancer development in vitro, we discerned that additional retinoic acid rescued the cell mortality caused by disulfiram (Supplementary Fig. S9A–E). This showed a similar outcome when mTORC1 was activated with MHY1485 (Fig. 5E, F and Supplementary Fig. S8C–F). Experiments conducted on cells overexpressing ALDH1A1 further supported this observation (Supplementary Fig. S9F). Moreover, western blot outcomes indicated that retinoic acid could partially revert the disulfiram-mediated decrease in phopho-p70S6K expression (Supplementary Fig. S9G, H). This modulation paralleled the effects of the mTORC1 activator MHY1485 (Fig. 5G and Supplementary Fig. S8G).

**Fig. 6:**
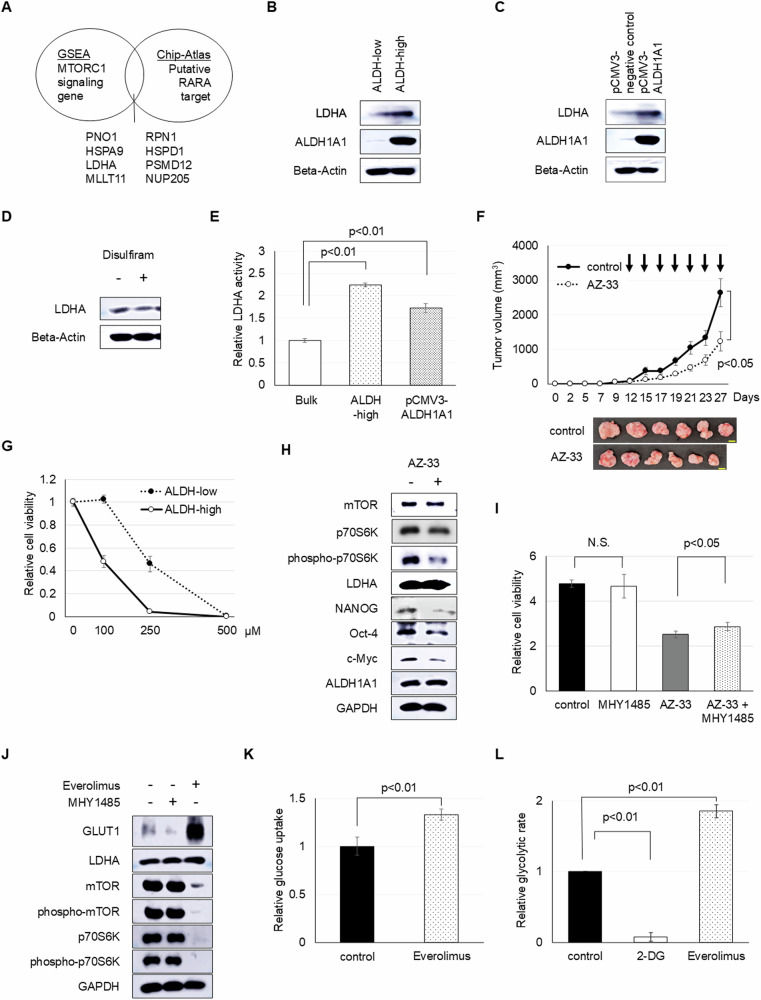
Interaction between glycolysis and mTOR controls the proliferation of ALDH-high endometrial cancer cells. See also Supplementary Figs. S9, S10, and S11. **A** In silico screening of RARA target genes connected to mTORC1 signaling using a combination of public chromatin immunoprecipitation (ChIP) database and gene set enrichment analysis based on microarray analysis of endometrial cancer spheroid cells. Combined with the 85 genes from 184 genes in the gene set of hallmark of mTORC1 signaling and putative RARA-regulated genes retrieved from ChIP-Atlas (http://chip-atlas.org/), eight genes were identified as the candidate genes correlated to ALDH-RARA-mTOR axis. Western blot analyses of **B** ALDH-high and ALDH-low spheroids cells, **C** the infected spheroids cells, and **D** disulfiram treatment. **E** Relative LDHA activity of control bulk, ALDH-high, and exogenous ALDH1A1 overexpressing spheroid cells. **F** Volumes (mean ± SEM) of xenograft tumors from 1 × 10^5^ EMN24 spheroid cells with the presence or absence of AZ-33 in vivo treatment. *n* = 6, Student’s *t*-tests. Xenograft tumor images post-excision on day 27 are on the right. Scale bar: 10 mm. **G** Relative ALDH-high and ALDH-low spheroid cell viability with the indicated in vitro AZ-33 treatment for 4 days. **H** Western blot analyses of spheroids cells after AZ-33 treatment for 24 h. **I** Relative spheroid cell viability in the presence or absence of 100 µM AZ-33 and/or 1 µM MHY1485 in vitro treatment for 4 days. **J** Western blot analyses of spheroids cells after everolimus or MHY1485 treatment for 24 h. **K** Relative glucose uptake of spheroid cells with 80 µM everolimus in vitro treatment. **L** Relative glycolytic rate of spheroid cells with 25 µM everolimus or 10 mM 2-DG in vitro treatment.

To assess the influence of RA on mTOR activation, we combined GSEA results from microarray data and published RARA binding data from the ChIP-Atlas (http://chip-atlas.org/). Of 184 hallmark mTORC1 signaling genes (Fig. 5B) [28], 85 were core enrichment genes associated with ALDH-high endometrial cancer cells. Merging this with RARA-regulated genes from ChIP-Atlas, specifically those linked to malignancies, yielded eight candidate genes (Fig. 6A). LDHA was predominantly expressed in ALDH-high cells (Fig. 6B). Further investigation revealed that ALDH1A1 overexpression increased LDHA levels, whereas ALDH inhibition, using disulfiram, decreased its expression (Fig. 6C, D and Supplementary Fig. S10A). Additionally, ALDH-high cells and exogenous ALDH1A1-overexpressing cells exhibited higher LDHA activity than control bulk spheroid cells (Fig. 6E). Meanwhile, Alpelisib and Ipatasertib treatment did not affect LDHA expression (Supplementary Fig. S10B, C).

Investigation of LDHA inhibition in cancer cells revealed that LDHA inhibitor AZ-33 considerably hampered endometrial cancer cell proliferation in vitro and in vivo (Fig. 6F and Supplementary Fig. S10D, E), especially in cells with ALDH activity (Fig. 6G). Although the LDHA inhibitor diminished LDH activity and phospho-p70S6K expression, ALDH expression and its functional activity remained unaltered in both bulk and exogenous ALDH1A1-overexpressing cells (Fig. 6H and Supplementary Fig. S10F). Furthermore, treatment with AZ-33 led to a marked reduction in stemness indicators such as Nanog, Oct-4, and c-Myc (Fig. 6H). In addition to AZ-33 treatment, LDHA knock down using RNAi decreased phospho-p70S6K expression and in vitro cell proliferation (Supplementary Fig. S10G, H). Additionally, activating mTORC1 with MHY1485 could partially counteract the inhibitory effects of AZ-33 on cancer cell proliferation, although MHY1485 in isolation did not make a significant difference (Fig. 6I and Supplementary Fig. S10I, J).

Collectively, our findings highlight that ALDH influences mTORC1 through LDHA, promoting the proliferation of endometrial cancer spheroid cells. Notably, although fluctuations in mTORC1 activation were independent of LDHA levels, the inhibition of mTOR with everolimus resulted in an increase in GLUT1 levels, influencing glucose transport and glycolytic rate, but not influencing LDHA expression (Fig. 6J–L and Supplementary Fig. S11A–D). Conversely, stimulating mTOR with MHY1485 reduced GLUT1 levels (Fig. 6J and Supplementary Fig. S11A), These intricate interplays underscore a reciprocal relationship between glycolysis and mTORC1 in ALDH-active endometrial cancer progression (Supplementary Fig. S12).

### LDHA expression is associated with adverse prognosis in endometrial cancer patients

**Fig. 7:**
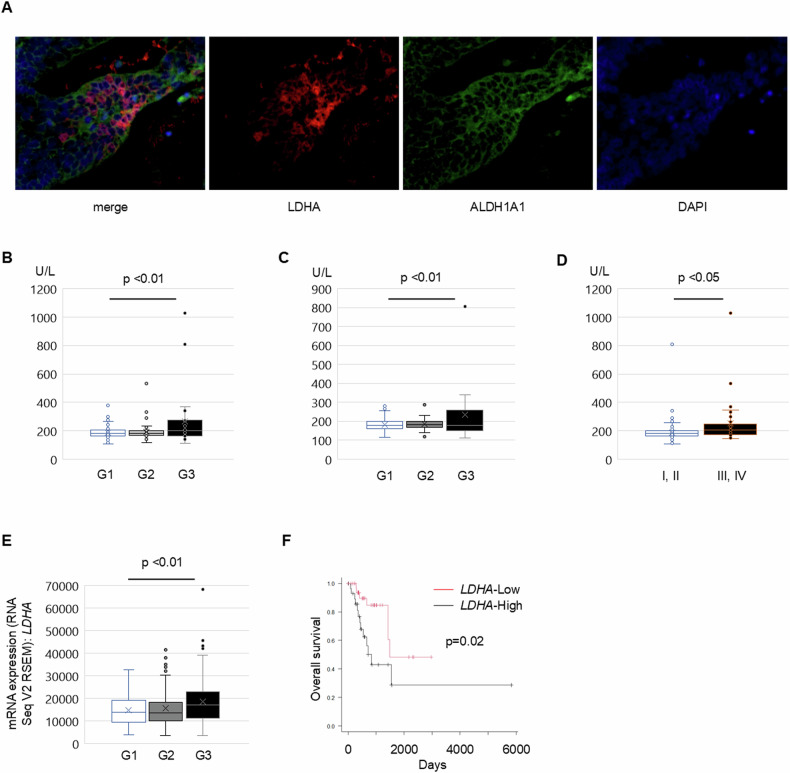
LDH is associated with clinical advanced stage and poor prognosis in endometrial cancer. See also Supplementary Fig. S13. **A** Combination staining of ALDH1A1 (green), LDHA (red), and DAPI (blue) in human endometrial cancer tumor samples. Serum LDH titer of the endometrial cancer patients before primary treatment **B** in different grades of all-clinical stage patients, **C** of advanced-stage patients, and **D** in different clinical stages of endometrial cancer. **E** *LDHA* mRNA level of endometrial cancer patients in different grades (TCGA database). **F** Overall survival of advanced-stage high-grade endometrial cancer patients with a high or low level of *LDHA* expression (TCGA database, black; *LDHA*-high, *n* = 29. Red; *LDHA*-low, *n* = 37; *p* = 0.02).

Our study suggests that glycolysis and mTORC1 signaling play a pivotal role in regulating ALDH-high endometrial cancer cells. Seeking to delineate the distribution of ALDH and LDHA in a clinical framework, we performed immunostaining analyses on endometrial cancer samples. ALDH-positive cells exhibited more pronounced LDHA expression than their ALDH-negative counterparts across both early and advanced cancer stages (Fig. 7A and Supplementary Fig. S13A).

To further elucidate the clinical implications of LDHA, serum LDH levels were quantified in 244 patients with endometrial cancer from our institution. Notably, LDH levels increased in patients with high-grade cancer relative to their low-grade counterparts across all stages, a trend particularly evident in stage I (Fig. 7B, C). Moreover, advanced-stage patients consistently exhibited elevated LDH titers in contrast to those in the early stages (Fig. 7D). This underscores a direct correlation between serum LDH levels, tumor grade, and clinical cancer progression.

Further exploring the clinical ramifications of LDHA expression, we utilized The Cancer Genome Atlas (TCGA) database [4] to examine its correlation with the prognosis of patients with endometrial cancer. *LDHA* mRNA levels were discernibly elevated in high-grade tumors (Fig. 7E). Patients manifesting elevated *LDHA* expression experienced a significantly reduced overall survival (Fig. 7F, *p* = 0.02). Moreover, progression-free survival showed a concerning trend, with *LDHA*-high-expressing cases displaying a tendency toward shorter survival compared with their low-expression counterparts (Supplementary Fig. S13B, *p* = 0.09). A significant correlation was also evident between *LDHA* and *RPS6KB1* expression in endometrial cancer tissue (Supplementary Fig. S13C, *p* = 0.01).

In summary, our findings highlight *LDHA* as a crucial biomarker, revealing its strong association with tumor grade and suggesting its potential role as an indicator of unfavorable prognosis in endometrial cancer.

## Discussion

The patient-derived cell model shows promise in understanding therapeutic responses to cytotoxic chemotherapeutic agents on an individual patient basis [29]. Recent findings suggest organoid cells can effectively serve as models for evaluating responses to therapies targeting specific genetic mutations [30, 31]. Consistent with these studies, the present study highlighted the correlation between in vitro and in vivo tests concerning the sensitivity to PI3K–Akt–mTOR signaling inhibitors in endometrial cancer patient-derived spheroid cells. In particular, the differentiation in response to PI3K and Akt inhibitors based on genomic mutation profiles highlights the clinical potential of spheroid cells. Conversely, it is possible that several factors, including the PI3K–Akt–mTOR pathway as well as *KRAS* or *TP53* mutations, are intricately linked in a complex response to the agents.

Endometrial, breast, and colon cancers often present activation in the mutant-dependent PI3K–Akt–mTOR signaling pathway [2, 4, 32]. Although PI3K–Akt–mTOR signaling inhibitors have theoretical potential to markedly suppress mutated cancer cells, challenges arise from negative feedback release and detrimental impacts on non-tumor cells. Furthermore, elements such as growth factors, energy, and stress play roles in mTOR activation [3]. This prompted our investigation into the non-genetic mechanisms underlying PI3K–Akt–mTOR pathway activation during endometrial cancer proliferation. The PI3K–Akt–mTOR pathway has been implicated in enhancing stemness, a trait associated with aggressive cancer manifestations [33]. Numerous studies have identified the role of PI3K signaling in cancer stemness, affecting markers such as Nanog, SOX2, and CD133 [34–36]. For instance, mTORC1 activation reportedly boosts colon cancer stem cell proliferation [37]. mTOR pathway is the downstream mediator of ALDH1A3 in gastric cancer [37]. Our studies on uterine endometrial cancer emphasized the influence of both genomic and ALDH-mediated non-genomic aberrations on mTOR activation. Spheroid cells, enriched with cancer stem cells, were integral in clarifying the relationship between cancer stemness and PI3K–Akt–mTORC1 signaling [38].

Lactate dehydrogenase (LDH), an intracellular enzyme, can manifest as either a homotetramer or heterotetramer, encompassing LDHA and LDHB subunits, giving rise to five isoforms. Each isoform demonstrates proficiency in the conversion of pyruvate to lactate [39]. Consistent with previous reports on cancers such as breast and pancreatic [40–42], our analysis corroborates the association between LDHA expression and advanced-stage endometrial cancer prognosis. Furthermore, elevated LDHA expression and serum LDH levels were more prevalent in high-grade cancer patients. Previous studies have identified correlations between elevated serum LDH levels and reduced survival rates in solid tumors such as melanoma and prostate carcinomas, highlighting its potential as a prognostic biomarker for metastatic carcinomas [43]. Collectively, these insights emphasize the role of LDHA in exacerbating endometrial cancer progression and its clinical malignancy.

Extensive research is currently focused on studying the relationship between glycolysis and cancer proliferation, particularly processes such as epithelial-to-mesenchymal transition and cancer stemness [44]. Our previous study revealed the centrality of the glucose transporter GLUT1 in dictating the stemness and chemoresistance traits of ALDH-active endometrial cancer stem cells [12]. We later discovered the regulatory role of ALDH-mediated retinoic acid over glycolytic functional factor LDHA. LDHA is crucial for sustaining breast cancer stemness and triggering metastasis [41]. Few studies have analyzed the role of LDHA in governing mTOR activation. In pancreatic adenocarcinoma, LDHA-induced proliferation is mediated by AMPK-mTOR signaling through L-lactate [40]. Other findings emphasize the role of LDHA in activating glycolysis, subsequently stimulating mTORC1 and thus fostering cellular proliferation. This phenomenon is particularly observed in K-Ras activated colon and pancreatic cancer cells [45]. Furthermore, LDHA is required for the activation of mTOR in gastric cancer [46]. Corroborating these findings, our research establishes that inhibiting LDHA impedes mTOR activation and restricts cancer cell proliferation, particularly in ALDH-active endometrial cancer spheroids, regardless of K-Ras genomic aberrations (Fig. 6).

Moreover, our findings reveal the role of mTOR in modulating glycolysis. Inhibiting Akt–mTORC1 signaling decreased GLUT1 expression, disrupting glycolysis and cancer cell survival in diseases such as leukemia [47, 48]. The intricate dynamics between mTOR and glycolysis in cancer encompass both direct and indirect mechanisms. A notable aspect of our study highlights the mutual regulatory relationship between glycolysis and mTOR activation. This feedback mechanism orchestrates the proliferation and survival of ALDH-active endometrial cancer stem cells (Fig. S12). Targeting this interaction could potentially enhance endometrial cancer treatment outcomes, even when mTORC1 activation is influenced by PI3K–Akt–mTOR genomic aberrations.

In summary, we revealed that the ALDH–LDHA–mTORC1 cascade represents a novel facet of the ALDH-related signaling that regulates the proliferation of uterine endometrial cancer stem cells. Future studies should further investigate the cascade and interaction between glycolysis and mTORC1 to develop clinical treatment strategies for addressing the progression of aggressive uterine endometrial cancer.

## Materials and methods

### Tumor-derived spheroid culture

Endometrial cancer spheroid cells isolated from clinical cancer specimens were cultured using ultra-low-attachment culture dishes (Corning, Corning, NY, USA). The culture medium was STEMPRO hESC SFM (Gibco, Grand Island, NY, USA) with a supplementation of 8 ng/ml basic fibroblast growth factor (Invitrogen, Carlsbad, CA, USA) and penicillin/streptomycin. Cells were maintained under specific conditions at 37 °C with 5% CO_2_ concentration [15]. Spheroid cells were dissociated using Accumax (Innovative Cell Technologies, San Diego, CA, USA) for serial passaging.

### Animal experiments

The drug efficacy experiments followed a randomized selection of mice into distinct groups (*n* = 6 or 8). Accumax (Innovative Cell Technologies) was used to dissociate spheroid cells into single-cell structures. These cells were then suspended in a medium, comprising 50% Matrigel (BD Biosciences, San Jose, CA, USA; 3564234), before being subcutaneously injected using a 27-G needle into NOG (NOD/Shi-SCID-IL-2Rγnull) mice. These mice were sourced from the Central Institute for Experimental Animals, Kawasaki, Japan. The drug was administered every 2–3 days from 10 to 20 days after spheroid cell injection. The control group was exposed to dimethyl sulfoxide (DMSO) only. Mice were monitored every 3–4 days for a span of 4–6 weeks post-cell transplantation.

### Lentivirus-mediated transduction

Both pCMV3-ALDH1A1 plasmid (HG11388-UT) and pCMV3 control vector were procured from Sino Biological (Beijing, China). The process of creating virus-containing supernatants and the subsequent viral infections were carried out as elaborated in a previously established protocol [11]. Post-infection, cells underwent selection in a medium containing 100 μg/ml hygromycin.

### Western blot analyses

The cell lysis procedure was facilitated using radioimmunoprecipitation assay buffer. This buffer contained specific concentrations of several compounds, including 50 mM Tris (pH 8.0), 150 mM NaCl, 1% Nonidet P-40, 0.5% sodium deoxycholate, 0.1% sodium dodecyl sulfate, and 1 mM ethylenediaminetetraacetic acid. To this mixture, protease and phosphatase inhibitors (Roche, Basel, Switzerland) were added. The resultant samples were then subjected to western blot analysis as detailed in a previous protocol [11], with the use of specific primary antibodies (Details provided in Supplementary Table 1).

### Statistical analyses

The statistical evaluation of both in vitro and in vivo spheroid cell experiments utilized Welch’s *t*-tests or Student’s *t*-tests, contingent on the outcomes of *F* tests. A threshold of *p* < 0.05 was set for significance. Clinical sample statistics were performed using EZR software [49]. The Kaplan–Meier method was used for univariate survival analysis, and the significance of variances between groups was determined through log-rank tests.

## Supplementary information

Supplementary Data (Supplementary Figure S1-S13, Supplementary Table Legends, and Supplementary Experimental Procedures)

Supplementary Table S1

Supplementary Table S2

original western blot figure

## Data Availability

The microarray data analyzed in this study were obtained from the Gene Expression Omnibus database (accession number: GSE123530). Additional materials and methods can be retrieved in Supplemental Experimental Procedures.
