# Supplementary material for: Glycolysis-mTORC1 crosstalk drives proliferation of patient-derived endometrial cancer spheroid cells with ALDH activity
Source: Cell Death Discov. 2024 Oct 11;10:435. doi: 10.1038/s41420-024-02204-y (PMC11470041; doi:10.1038/s41420-024-02204-y)
Supplement: Supplementary file 1 — Supplementary Data (Supplementary Figure S1-S13, Supplementary Table Legends, and Supplementary Experimental Procedures) [file 41420_2024_2204_MOESM1_ESM.docx]

**Supplementary Data**

**Supplementary Figure Legends**

**Fig. S1. Aberrant expression of PI3K-Akt-mTOR signaling in endometrial cancer. Related to Figure 1.**

Immunohistochemical analysis of spheroid cells, primary endometrial cancer tumors and spheroid-derived xenograft tumors.


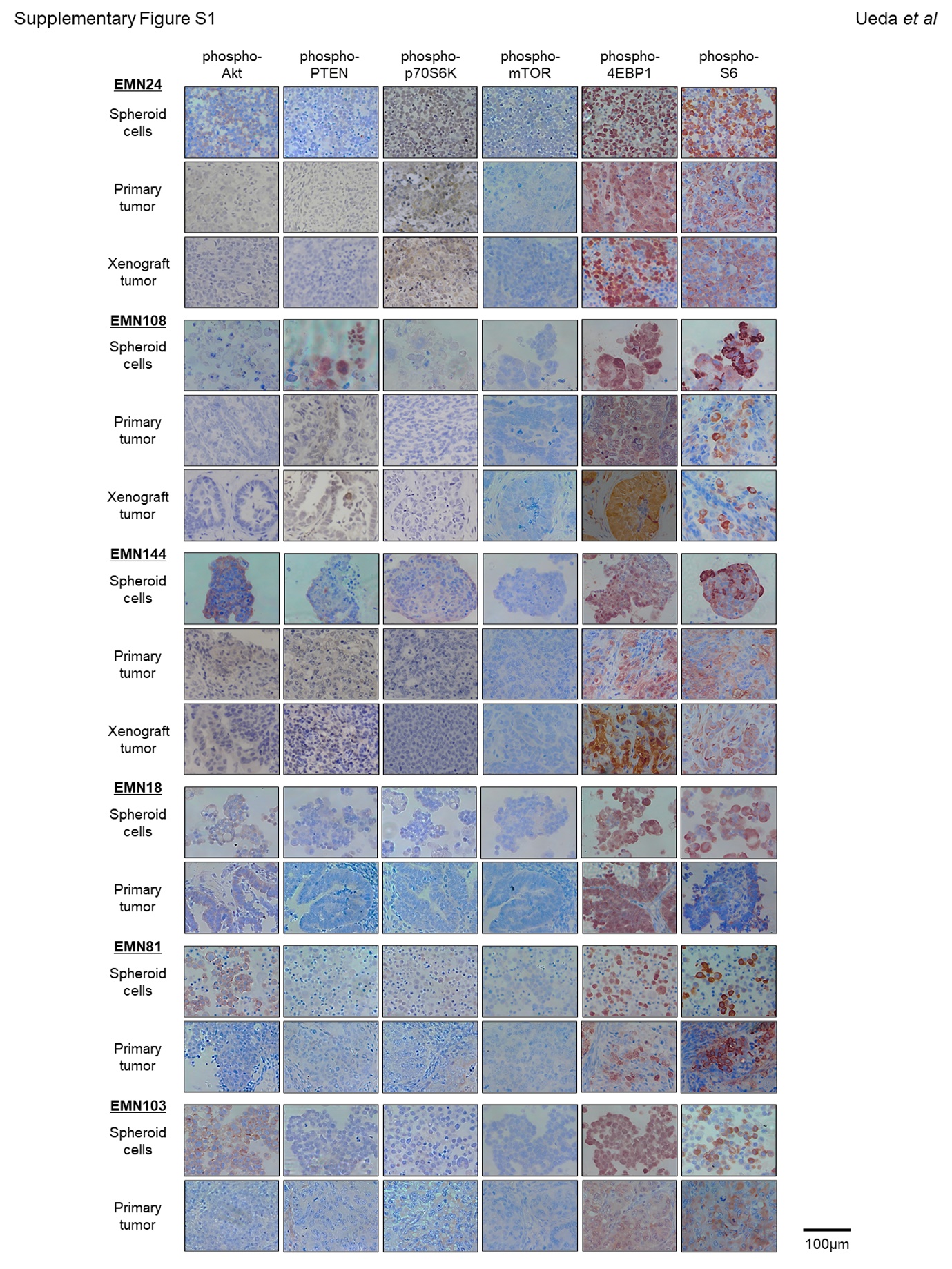


**Fig. S2. Clinical significance of phospho-p70S6K expression in endometrial cancer. Related to Fig. 1.**

(A), Kaplan–Meier plots of progression-free survival in advanced-stage endometrial cancer patients stratified by phospho-p70S6K positivity (red, *n =* 13) and negativity (black, *n =* 22). (B-C), Distribution of phospho-p70S6K expression in patients with advanced-stage endometrial cancer (*n =* 35) according to (B) tumor grade and (C) clinical stage.
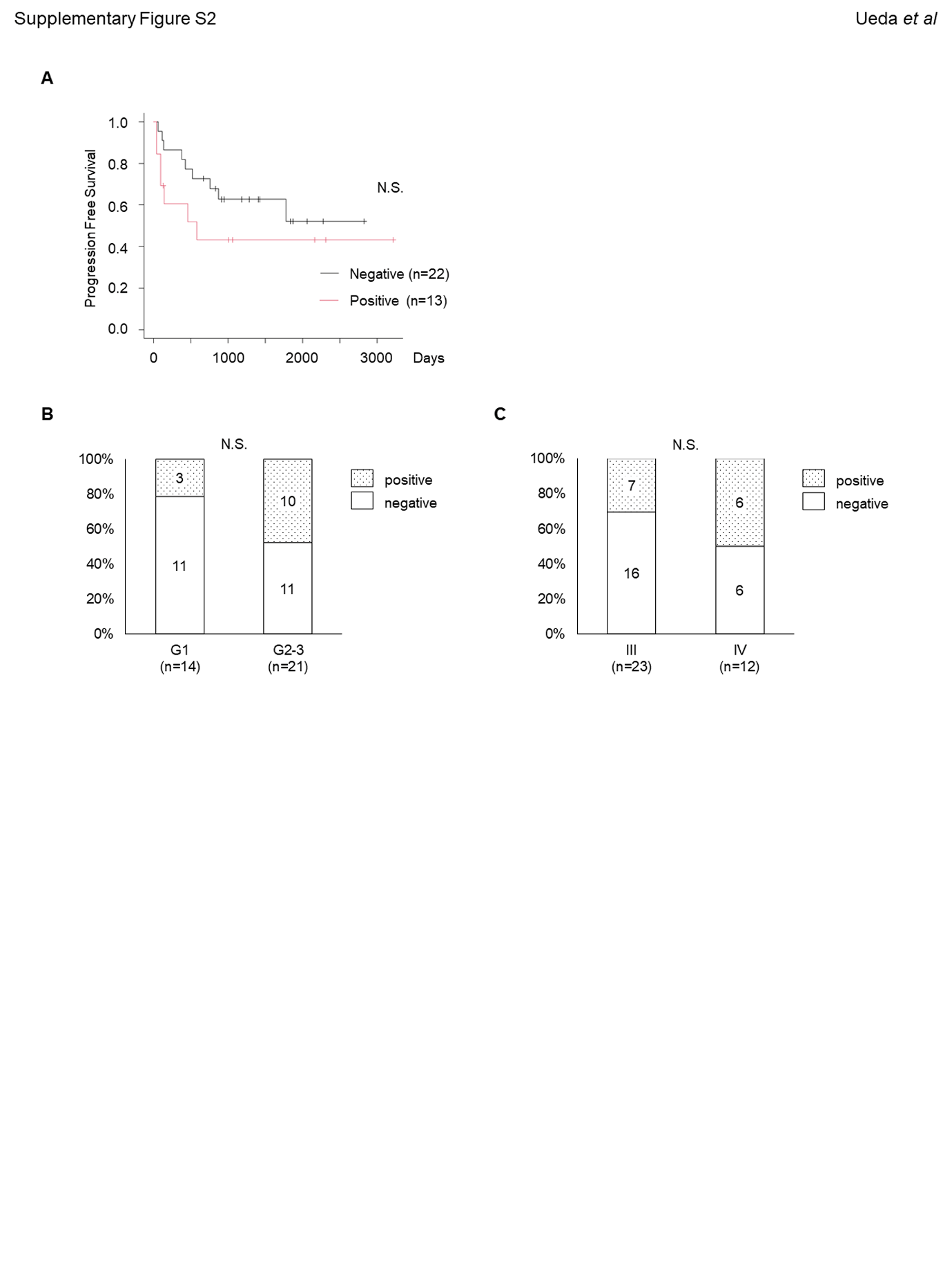


**Fig. S3. PI3K inhibitors block the formation and proliferation of spheroid cells. Related to Fig. 2.**

(A-B) Responses of spheroid cells to different concentrations of PI3K inhibitor after incubation for 4 days. (A) Taselisib and (B) Copanlisib. (C-D), Tumor volumes (mean ± SEM) of xenograft tumors from 5 × 10^5^ spheroid cells subcutaneously injected. Alpelisib (15 mg/kg) was intraperitoneally injected into mice in the Alpelisib-treatment group, and vehicle (DMSO) was intraperitoneally injected into mice in the control group. *n =* 6 or 8 independent experiments. (C) EMN81 cells, and (D) EMN144 cells, Student’s *t*-test.

**
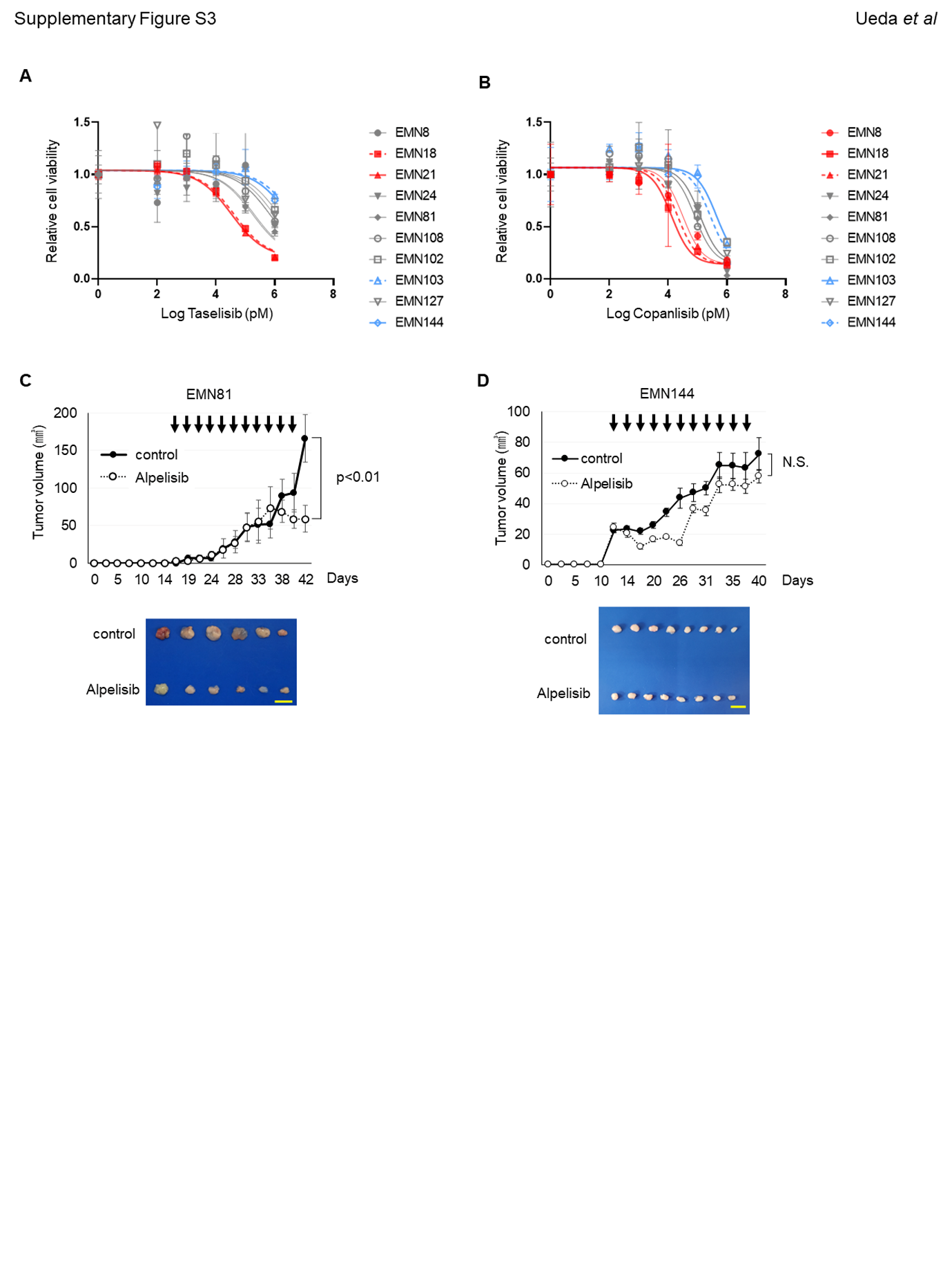
**

**Fig. S4. Combination therapy with Alpelisib and the ALDH inhibitor inhibits endometrial cancer cell progression. Related to Fig. 2.**

(A) FACS analysis of ALDH activity in EMN21 spheroid cells treated with Alpelisib for 4 days *in vitro*. (B) Western blot analyses of ALDH-high and ALDH-low spheroid cells in the presence or absence of Alpelisib treatment. (C) Volumes (mean ± SEM) of xenograft tumors from 5 × 10^5^ EMN24 spheroid cells with the presence or absence of Alpelisib (15 mg/kg) and/or disulfiram (40 mg/kg) after *in vivo* treatment. *n =* 8, Student’s *t*-tests. Images of whole resected tumor xenograft tumors excised on day 42 (bottom). Scale bar, 10 mm. (D) FACS of ALDH activity *in vivo*, post-treatment with combination of Alpelisib and disulfiram.


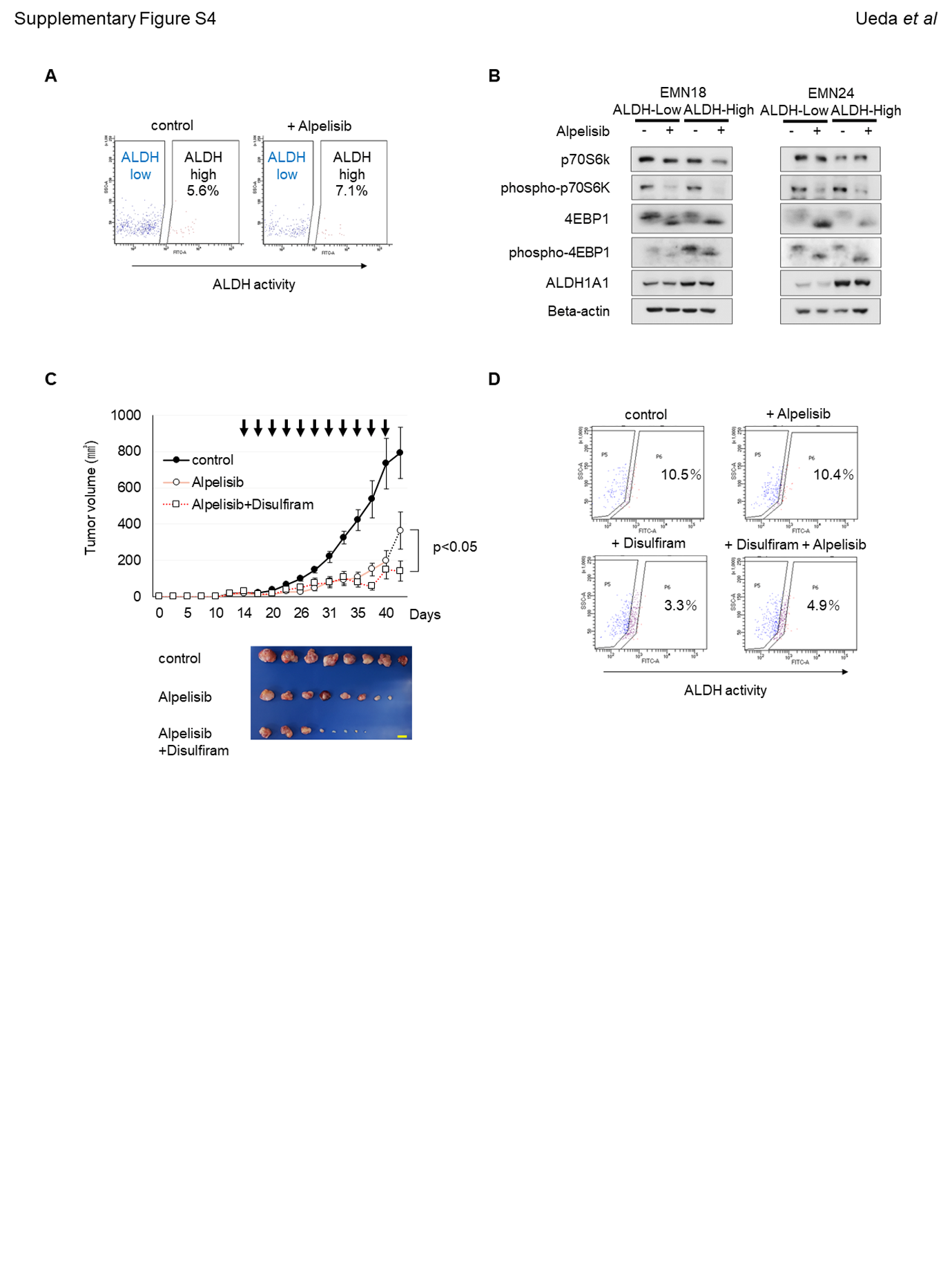


**Fig. S5. Ipatasertib treatment for endometrial cancer cell progression. Related to Fig. 3.**

Volume (mean ± SEM) of xenograft tumors from 1 × 10^5^ EMN24 cells were treated with or without ipatasertib (15 mg/kg) *in vivo*. *n =* 8, Student’s *t*-test.


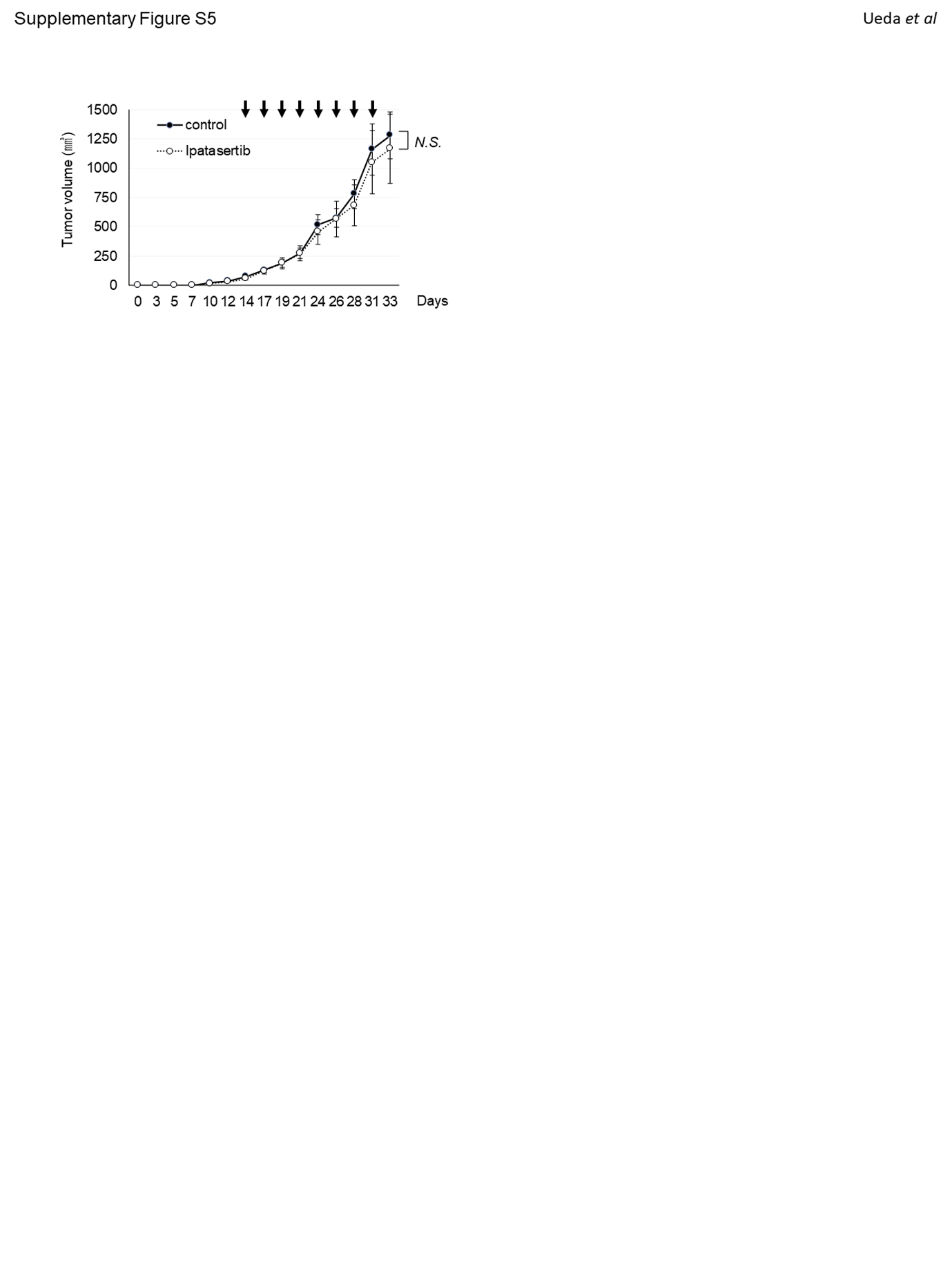


**Fig. S6. mTORC inhibitor treatment for endometrial cancer spheroid cells. Related to Fig. 4.**

(A) Responses of spheroid cells to different concentrations of Torin1 after incubation for 4 days. B, Western blotting analysis of ALDH-high and ALDH-low spheroid cells in the presence or absence of Torin1 (EMN24).


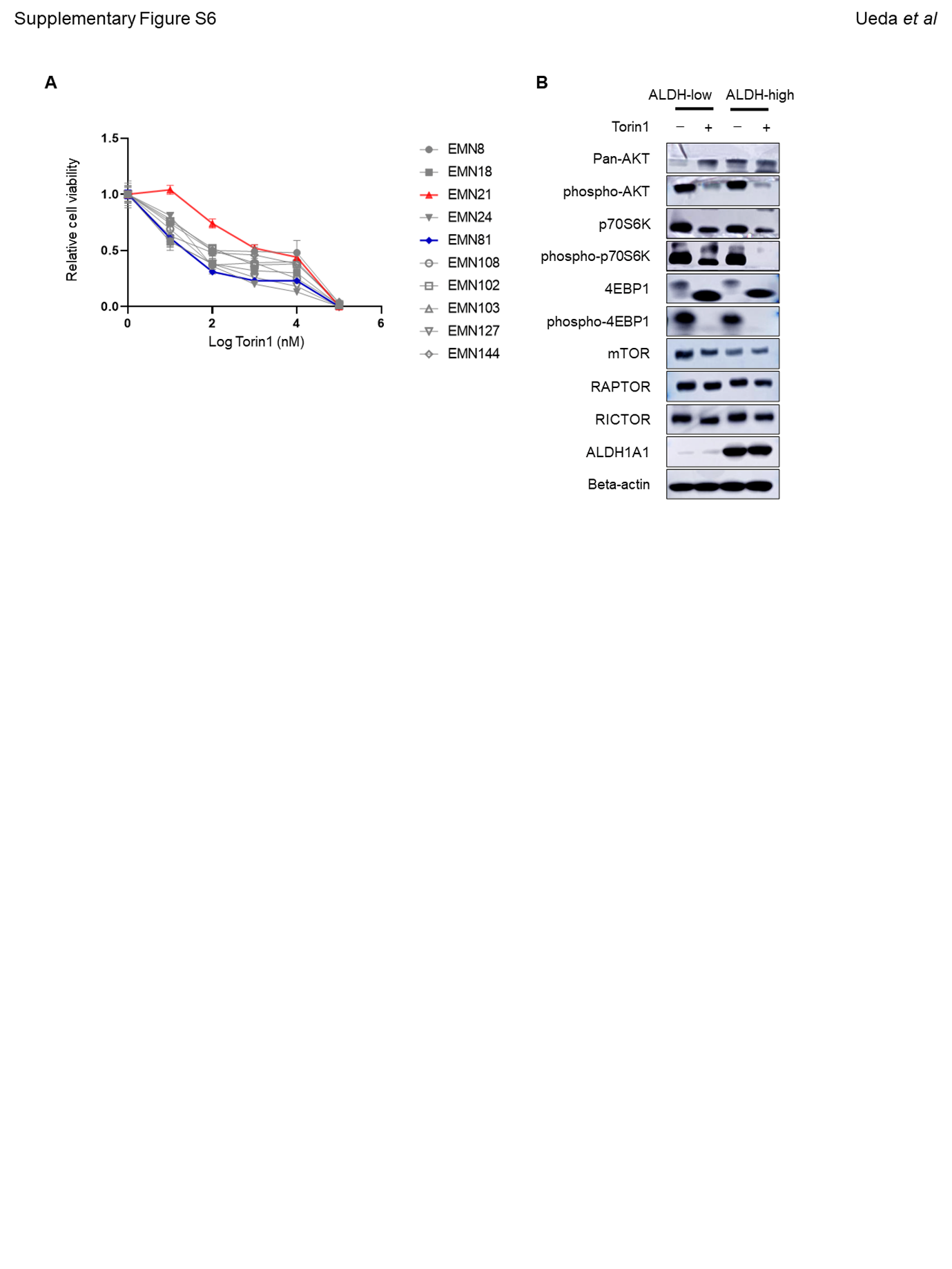


**Fig. S7. mTOR inhibitor blocks the proliferation of endometrial cancer spheroid cells (EMN21 cells). See also Supplementary Fig. 4.**

(A) Relatively infected spheroid cell viability with the indicated Everolimus *in vitro* treatment for 4 days. (B) Western blot analysis of the indicated spheroid cells.

**
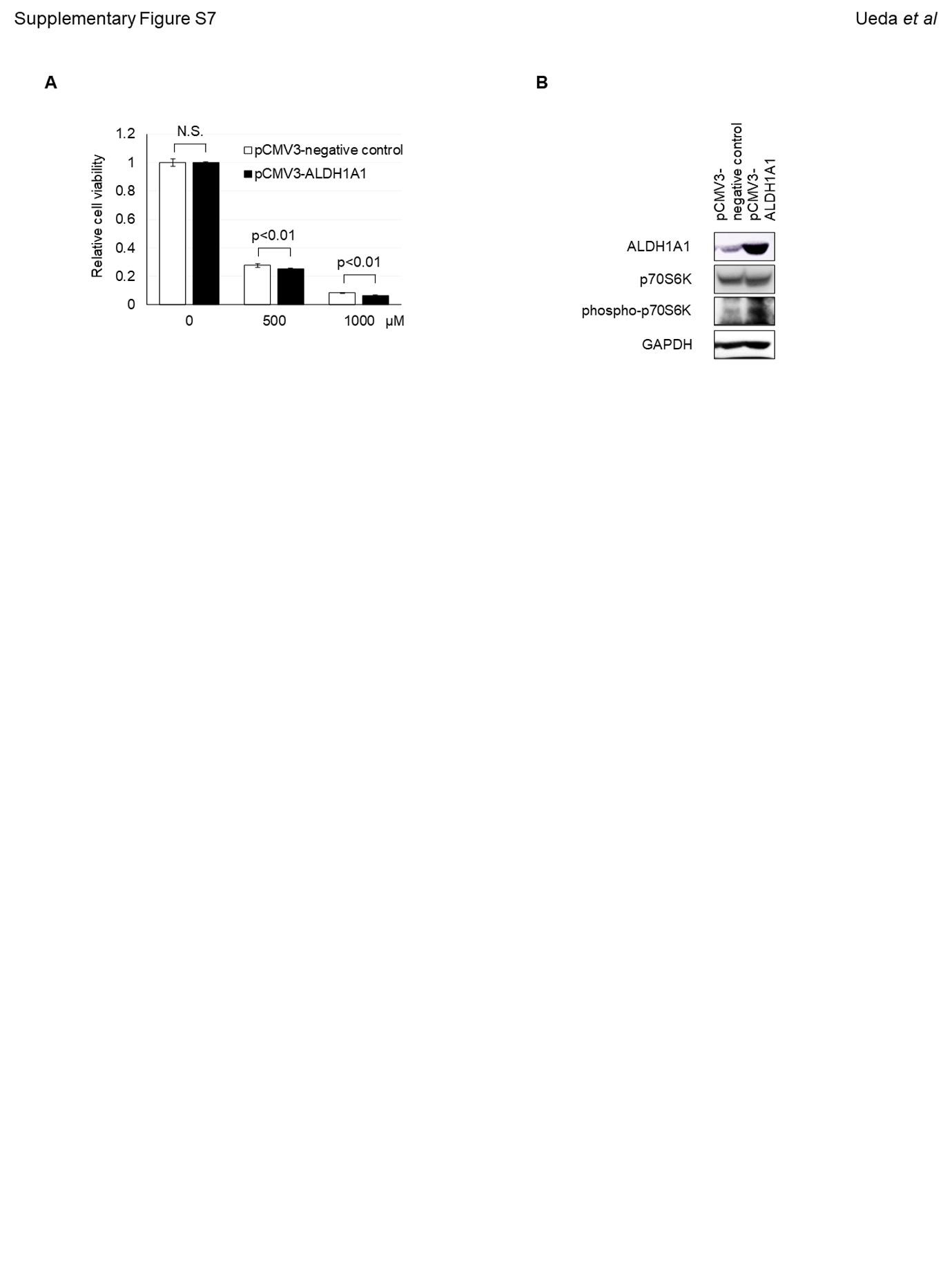
**

**Fig. S8. ALDH inhibitor blocks the proliferation of endometrial cancer cells via mTOR activity. Related to Fig. 5.**

(A) Western blot analysis of spheroid cells after disulfiram treatment for 24 h. (B) Gene set enrichment analyses of gene expression profiles of ALDH-high and ALDH-low cells (SIRNA_EIF4EGI_UP; human gene set of genes upregulated in control cells compared with eIF4GI silenced cells (left), and EIF4E_UP; human gene set of genes upregulated after ectopic expression of eIF4E (right)). (C-F) Relative spheroid cell viability in the presence or absence of disulfiram and/or MHY1485 *in vitro* treatment for 4 days. (C) EMN18, (D) EMN21, (E) EMN108, and (F) EMN144. (G) Western blot analysis of EMN144 spheroid cells after treatment with disulfiram and/or MHY1485 for 24 h.


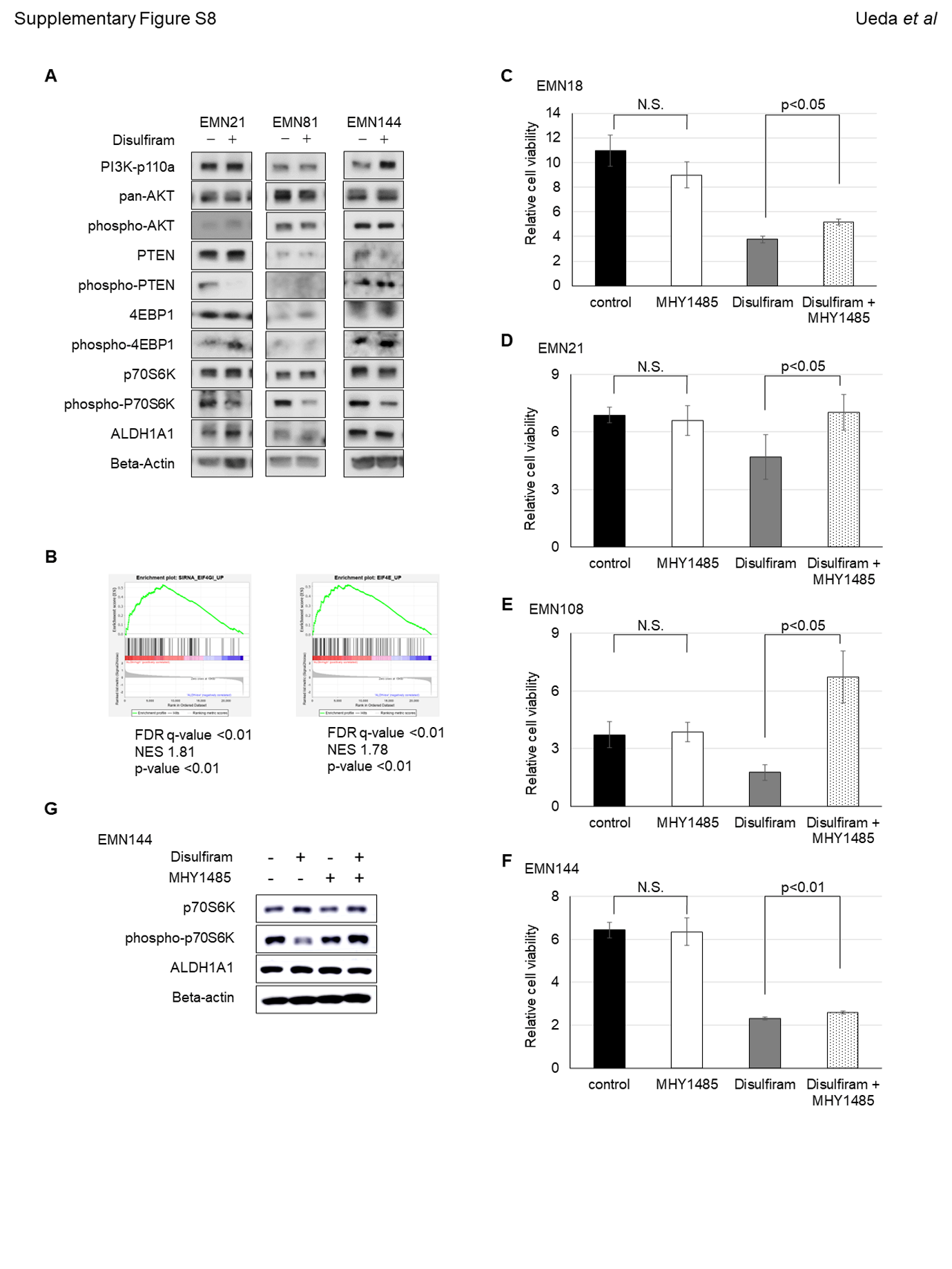


**Fig. S9. ATRA contributed to the proliferation of endometrial cancer spheroid cells. See also Fig. 6.**

(A-F) Relative spheroid cell viability in the presence or absence of disulfiram and/or ATRA *in vitro* treatment for 4 days. (A) EMN24 cells, (B) EMN144 cells, (C) EMN18 cells, (D) EMN108 cells, (E) EMN103 cells, and (F) exogenous ALDH1A1-expressing EMN24 cells. (G-H) Western blot analyses of spheroids cells after disulfiram and/or ATRA treatment for 24 h. (G) EMN144 cells and (H) EMN 24 cells.


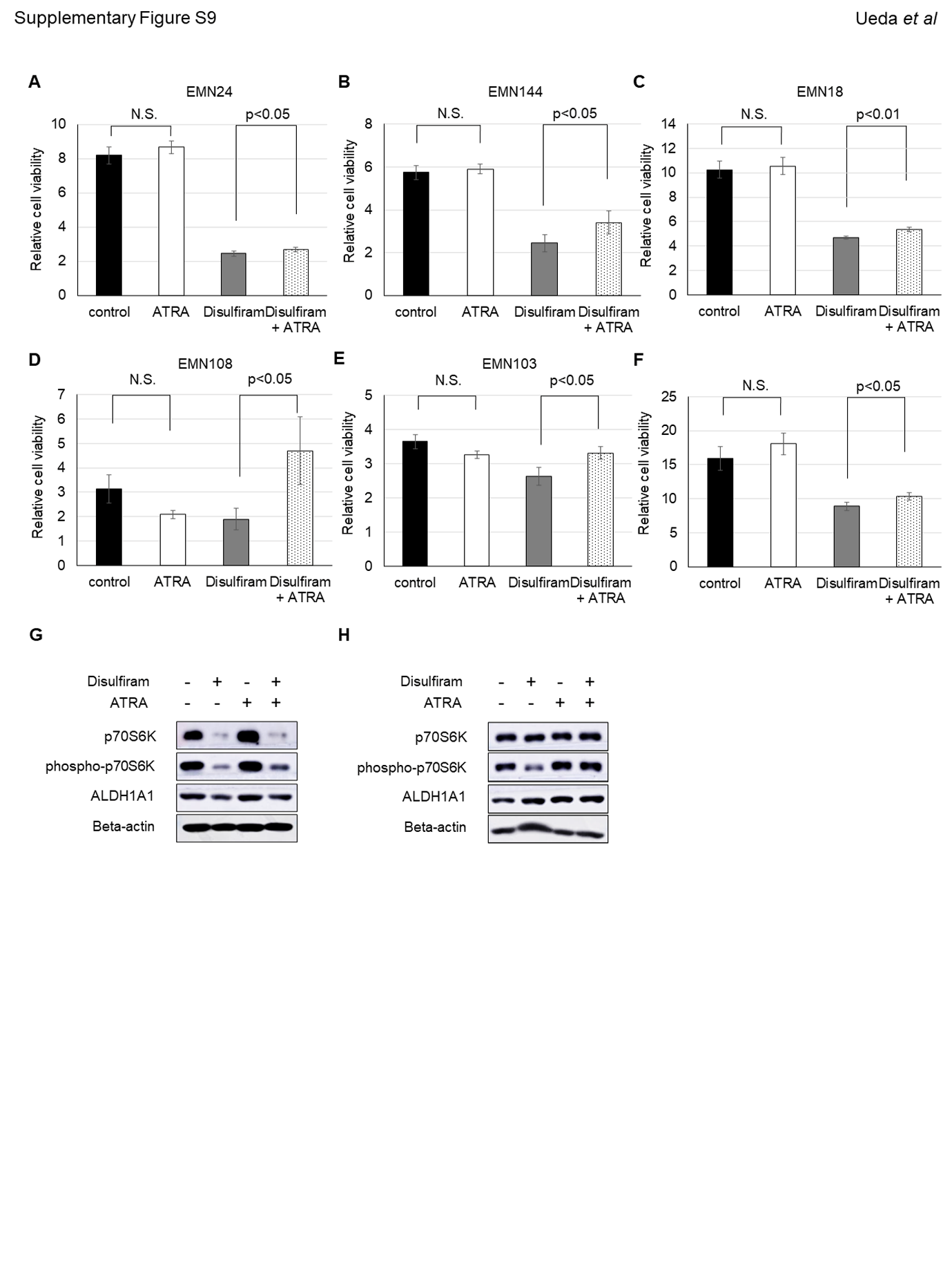


**Fig. S10. Interaction between glycolysis and mTOR controls the proliferation of ALDH-high endometrial cancer cells. See also Fig. 6.**

(A-C) Western blot analyses of spheroids cells with (A) Disulfiram, (B) Alpelisib, and (C) Ipatasertib treatment. (D) Volumes (mean ± SEM) of xenograft tumors from 1 × 10^5^ EMN108 spheroid cells with the presence or absence of AZ-33 *in vivo* treatment. *n =* 8, Student’s *t*-tests. Images of the whole resected tumor xenograft tumors excised on day 27 (bottom). Scale bar, 10 mm. (E) Time course of EMN24 spheroid cell proliferation in the presence or absence of AZ-33 treatment *in vitro* for 7 days. (F) Western blot analyses of exogenous ALDH1A1-expressing EMN24 spheroids cells with AZ-33 treatment for 24h. (G) Western blot analyses and (H) time course of the proliferation of indicated spheroids cells transfected with LDHA siRNA or control siRNA. (I-J) Relative spheroid cell viability in the presence or absence of AZ-33 and/or MHY1485 *in vitro* treatment for 4 days. (I) EMN18 and (J) EMN108 cells.


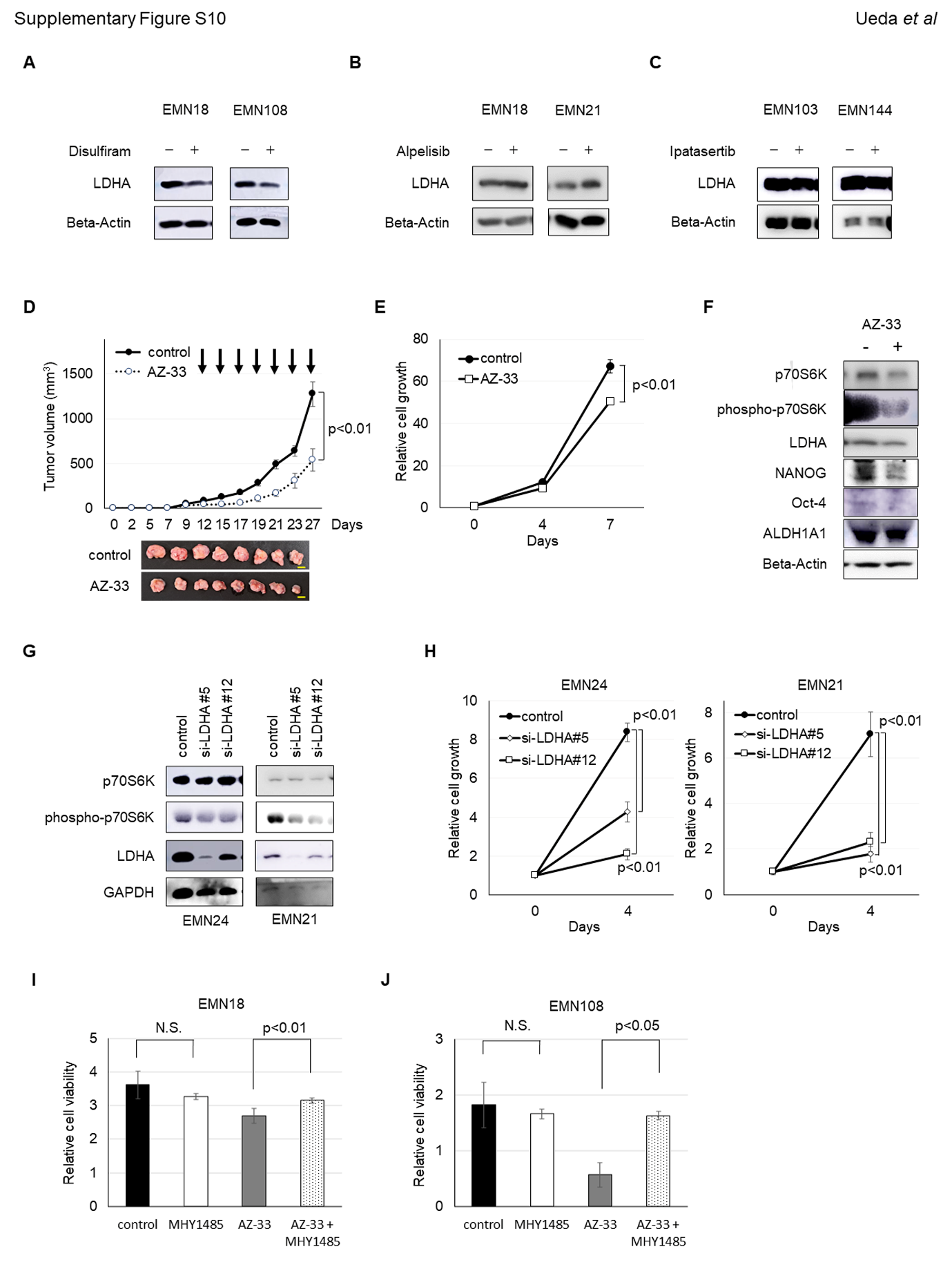


**Fig. S11. Interaction between glycolysis and mTOR controls the proliferation of ALDH-high endometrial cancer cells. See also Fig. 6.**

(A) Western blot analyses of spheroids cells after everolimus or MHY1485 treatment for 24 h. (B-C) Relative glucose uptake of spheroid cells in the everolimus *in vitro* treatment. (B) EMN18 and (C) EMN103 cells. (D) Relative glycolytic rate of EMN18 spheroid cells with 25 µM Everolimus treatment *in vitro*.

**
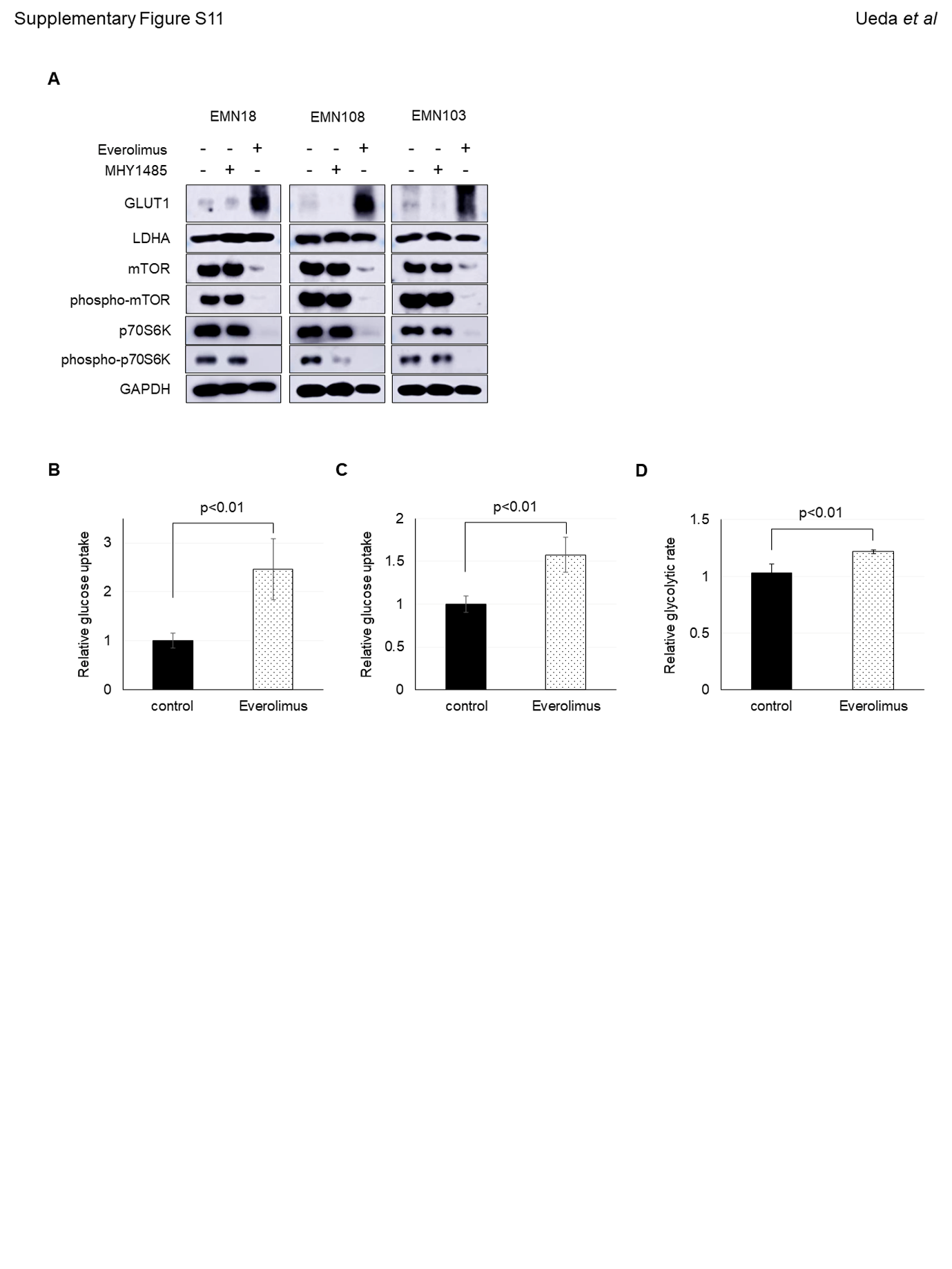
**

**Fig. S12. A model of glycolysis and mTOR regulation for the propagation of endometrial cancer spheroid cells with ALDH activity.**


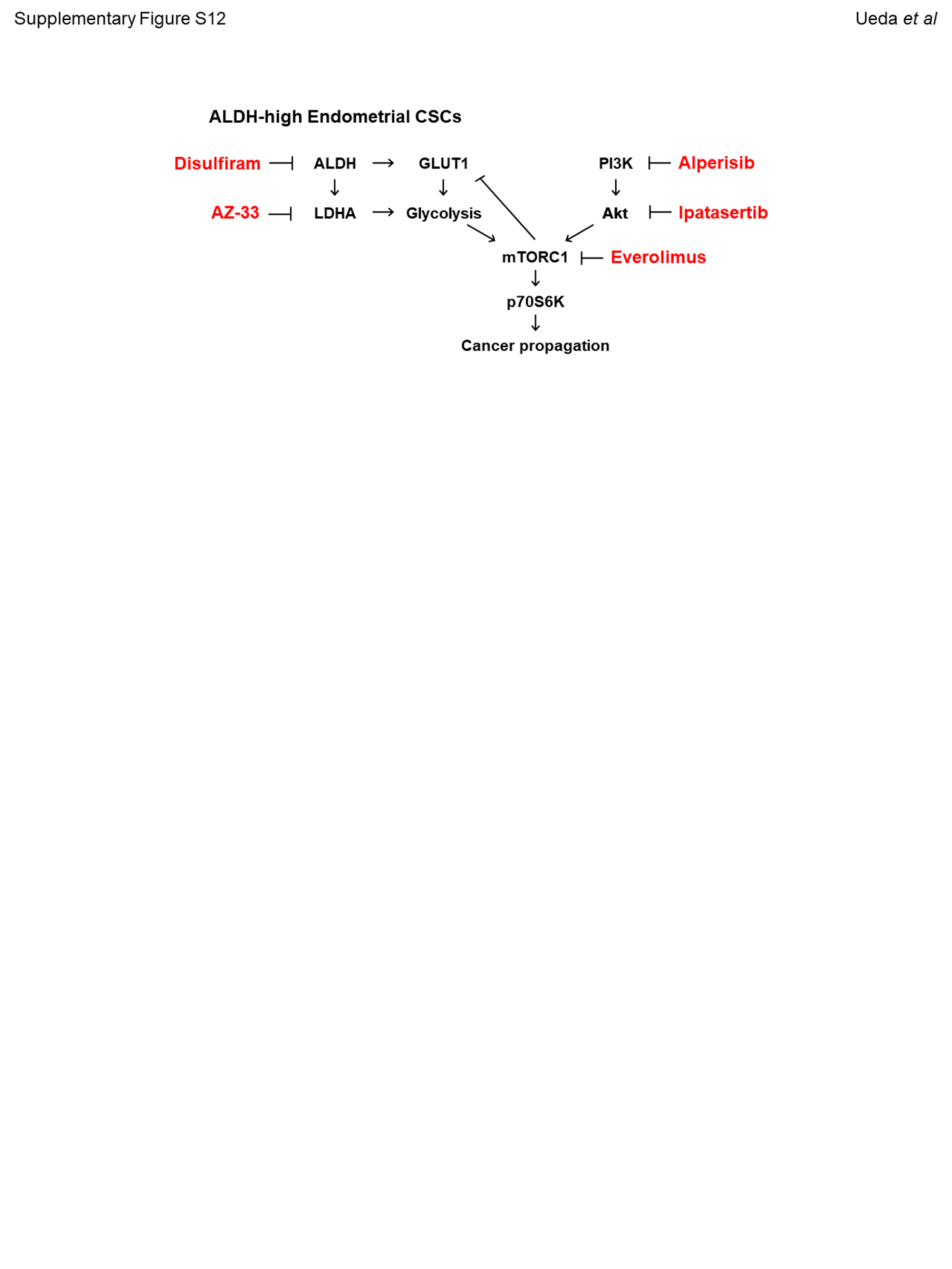


**Figure S13. Expression of LDHA in clinical endometrial cancer tumors. See also Figure 7.**

(A) Combined staining of ALDH1A1 (green), LDHA (red), and DAPI (blue) in human endometrial cancer tumor samples. (B) Progression-free survival of patients with advanced-stage, high-grade endometrial cancer with high or low *LDHA* expression (The Cancer Genome Atlas [TCGA] database, black; LDHA-high, *n =* 17). Red; LDHA-low, *n =* 36. *p* = 0.09). (C) Correlation between *LDHA* and *RPS6KB1* mRNA expression in endometrial cancer tumors (TCGA database, *n =* 177. *p* = 0.01).


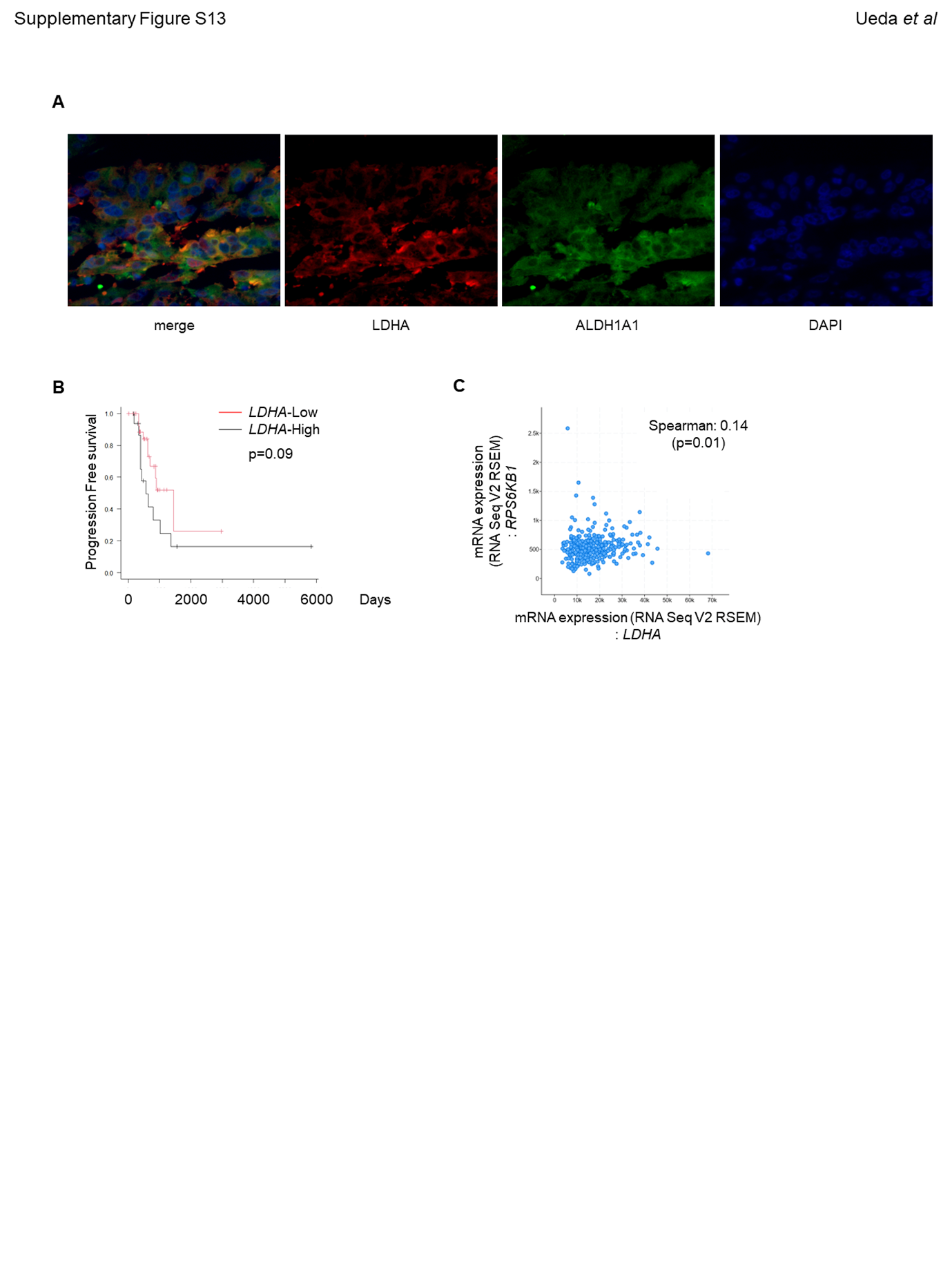
**Supplementary Table Legends**

**Table S1. List of gene mutations of spheroid cells and primary tumors**

**Table S2. List of reagents and resources used in this study**

**Supplemental Experimental Procedures**

*Targeted sequencing analysis*

Genomic DNA was extracted from both patient-derived frozen tumor tissues and spheroid cells. The targeted sequencing analysis employed the SureSelect NCC Oncopanel v4 (Agilent Technologies, Santa Clara, CA, USA), which captures the entire coding exons of 114 genes and the translocated introns of 12 genes (1). Sequencing libraries were constructed using the SureSelect XT Reagent Kit (Agilent Technologies) (2). We conducted paired-end sequencing (2 × 150 bp) with the NextSeq 500 (Illumina, San Diego, CA, USA). The Clinical Sequence Call system was utilized to detect mutations (single-nucleotide variations and short insertions/deletions), gene amplifications, and gene fusions (3).

*Gene set enrichment analysis (GSEA)*

Microarray analyses were conducted using Agilent Whole Human Genome 8 × 60 K oligo microarrays. This microarray data was sourced from the Gene Expression Omnibus database (accession number: GSE123530). We performed GSEA using the gene set collection version 6.2 from the Molecular Signatures Database (4, 5).

*Flow cytometry analyses*

Single spheroid cells were dissociated, filtered, and then incubated with 7-AAD (BD Pharmingen, Franklin Lakes, NJ, USA) to exclude nonviable cells, which were then used for ALDEFLUOR assays (Stem Cell Technologies, Vancouver, BC, CAN). Xenograft tumors underwent an enzymatic dissociation, and the resultant cancer cells were purified with the Mouse Cell Depletion Kit (Miltenyi Biotec, San Jose, CA, USA). The samples were subsequently analyzed and sorted using the FACSAria II and III Cell Sorter (BD Biosciences, San Jose, CA, USA).

*Cell-based assay*

We quantified cell viability using the CellTiter-Glo Luminescent Cell Viability Assays (Promega, Madison, WI, USA). The caspase-3/7 activity was determined using Caspase-Glo 3/7 3D Assay System (Promega). LDH activity and intracellular lactate levels were examined using the Lactate Dehydrogenase Activity Assay Kit (Merck, Kenilworth, NJ, USA) and Lactate-Glo™ Assay (Promega) following the manufacturer's protocols. Luminescence intensity was measured using the FLUOROSCAN instrument (Thermo Fisher Scientific, Waltham, MA, USA). Glycolytic rate was measured using Lactate-Glo™ Assay (Promega) following the manufacturer’s protocol. Briefly, cells were cultivated in glucose-free medium containing Everolimus, 2-deoxyglucose, or no compound. After treatment, glucose was added and incubated for 1h. Luminescence intensity was measured using the FLUOROSCAN instrument.

All *in vitro* cell-based assays were conducted in triplicate or quadruplicate, with results expressed as mean ± SD. No blinding was done.

siRNA transfection

siRNAs corresponding to LDHA was purchased from Qiagen; LDHA siRNAs (si#5: GeneGlobe ID: SI00300622, si#12: GeneGlobe ID: SI04949609) and control siRNA. From each, 50 nM siRNA were transfected into dissociated spheroid cells using the Xfect RNA transfection reagent (Takara, Japan) following to the manufacturer’s protocol.

*Immunohistochemical and immunofluorescence staining analyses*

Primary and mouse xenograft tumors were fixed in neutral formalin, paraffin-embedded, and subsequently stained with hematoxylin and eosin. For immunostaining, sections were processed using standard immunohistochemical (IHC) methods as detailed elsewhere (8). We utilized primary antibodies (refer to Table S1) and biotinylated secondary antibodies (Vector Laboratories, Burlingame, CA, USA). This was followed by incubation with ABC reagent (Dako, Glostrup, Denmark), followed by 3,3′-diaminobenzidine (Sigma-Aldrich, St. Louis, MO, USA). For immunofluorescence staining, sections were treated with a conjugated antibody and counterstained with 4′,6-diamidino-2-phenylindole.

During the staining evaluation, three randomized sample areas were assessed by two independent observers. Staining extent was visually gauged, and samples showing phospho-p70S6K staining in a minimum of 10% of the cancer cells were classified as IHC-phospho-p70S6K-positive. In contrast, samples with no staining or less than 10% staining were categorized as IHC- phospho-p70S6K-negative (Fig. 1C).

**Supplemental References**

1. Mori Y, Yamawaki K, Ishiguro T, Yoshihara K, Ueda H, Sato A, *et al.* ALDH-dependent glycolytic activation mediates stemness and paclitaxel resistance in patient-derived spheroid models of uterine endometrial cancer. *Stem Cell Rep* **2019**;13:730–46.

2. Asano N, Yoshida A, Mitani S, Kobayashi E, Shiotani B, Komiyama M, *et al.* Frequent amplification of receptor tyrosine kinase genes in welldifferentiated/ dedifferentiated liposarcoma. *Oncotarget* **2017**;8:12941–52.

3. Kato M, Nakamura H, Nagai M, Kubo T, Elzawahry A, Totoki Y, *et al.* A computational tool to detect DNA alterations tailored to formalin-fixed paraffin-embedded samples in cancer clinical sequencing. *Genome Med* **2018**;10:44.

4. Subramanian A, Tamayo P, Mootha VK, Mukherjee S, Ebert BL, Gillette MA, *et al.* Gene set enrichment analysis: a knowledge-based approach for interpreting genome-wide expression profiles. *Proc Natl Acad Sci U S A* **2005**;102:15545–50.

5. Mootha VK, Lindgren CM, Eriksson KF, Subramanian A, Sihag S, Lehar J, *et al.* PGC-1alpha-responsive genes involved in oxidative phosphorylation are coordinately downregulated in human diabetes. *Nat Genet* **2003**;34:267–73.

6. Di Veroli GY, Fornari C, Wang D, Mollard S, Bramhall JL, Richards FM, *et al.* Combenefit: an interactive platform for the analysis and visualization of drug combinations. *Bioinformatics* **2016**;32:2866–8.

7. Hu Y, Smyth GK. ELDA: extreme limiting dilution analysis for comparing depleted and enriched populations in stem cell and other assays. *J Immunol Methods* **2009**;347:70–8.

8. Ishiguro T, Sato A, Ohata H, Ikarashi Y, Takahashi RU, Ochiya T, *et al.* Establishment and characterization of an in vitro model of ovarian cancer stem-like cells with an enhanced proliferative capacity. *Cancer Res* **2016**;76:150–60.
