# Supplementary material for: Glycolysis-mTORC1 crosstalk drives proliferation of patient-derived endometrial cancer spheroid cells with ALDH activity
Source: Cell Death Discov. 2024 Oct 11;10:435. doi: 10.1038/s41420-024-02204-y (PMC11470041; doi:10.1038/s41420-024-02204-y)

Original Data Figure 1A



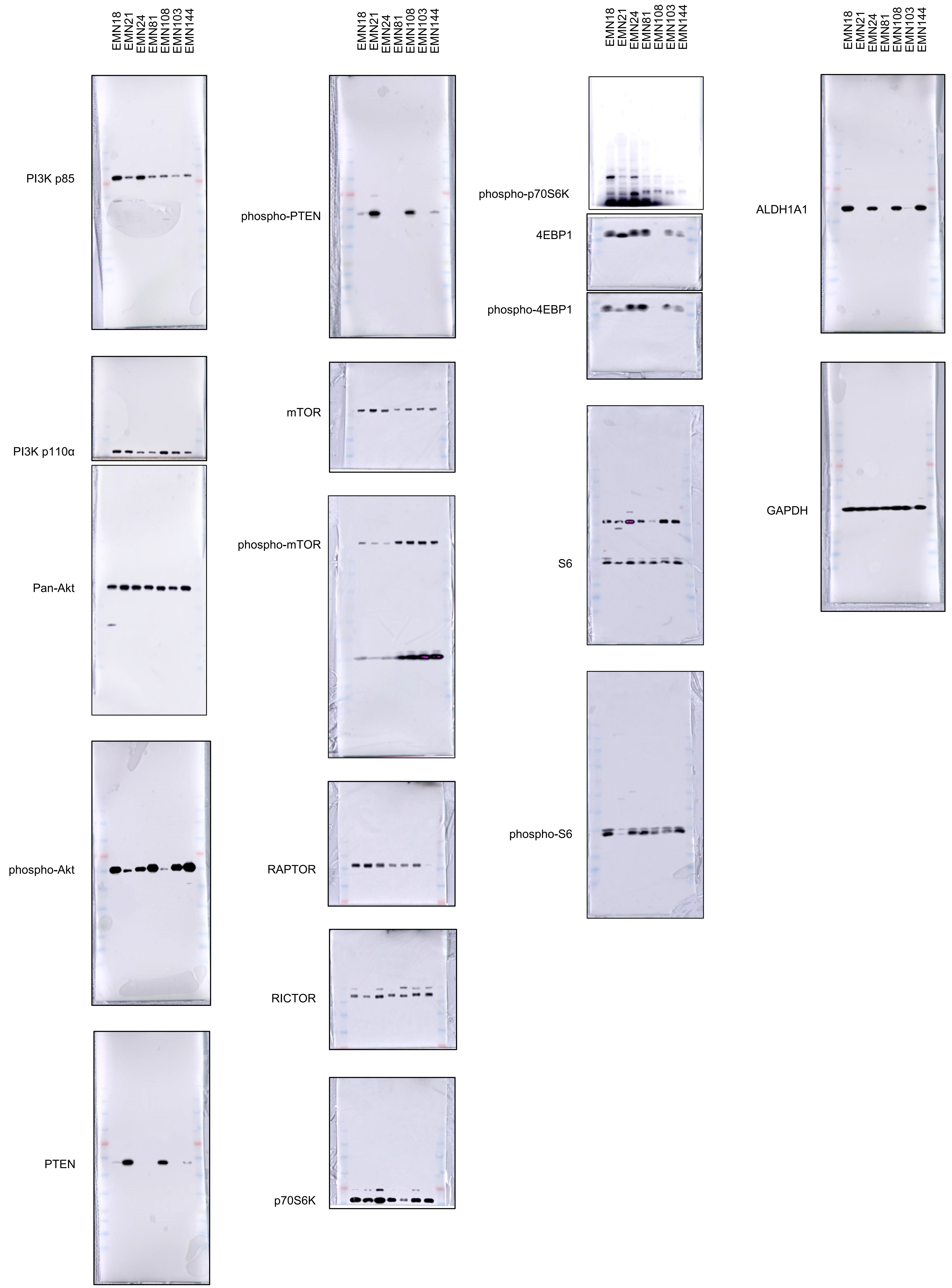

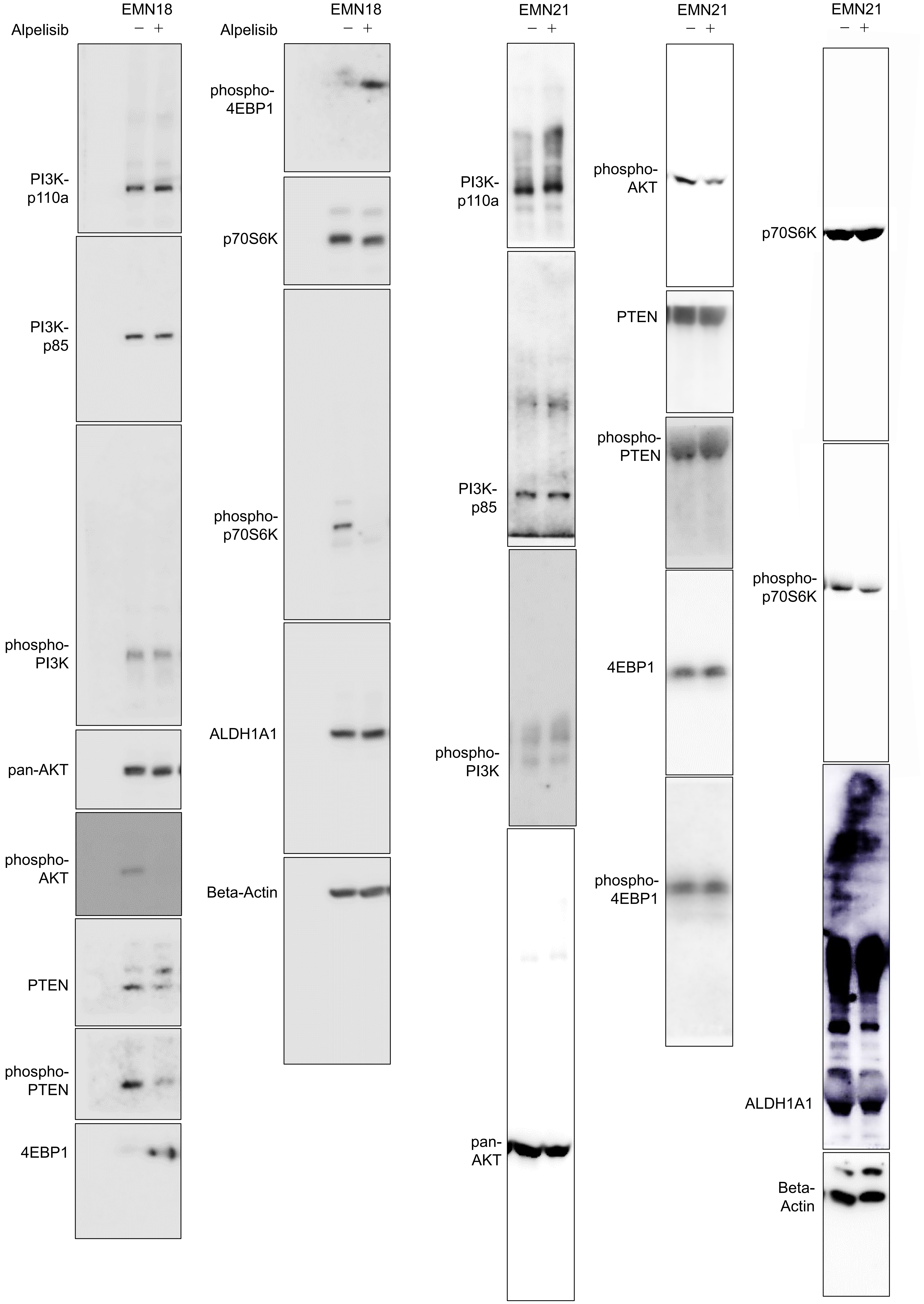
EMN18
Alpelisib
– +
PI3K-p110a
PI3K-p85
phospho-PI3K
pan-AKT
phospho-AKT
PTEN
phospho-PTEN
4EBP1
EMN18
Alpelisib
– +
phospho-4EBP1
p70S6K
phospho-p70S6K
ALDH1A1
Beta-Actin
EMN21
– +
PI3K-p110a
PI3K-p85
phospho-PI3K
pan-AKT
EMN21
– +
phospho-AKT
PTEN
phospho-PTEN
4EBP1
phospho-4EBP1
EMN21
– +
p70S6K
phospho-p70S6K
ALDH1A1
Beta-Actin

Original Data Figure 3E



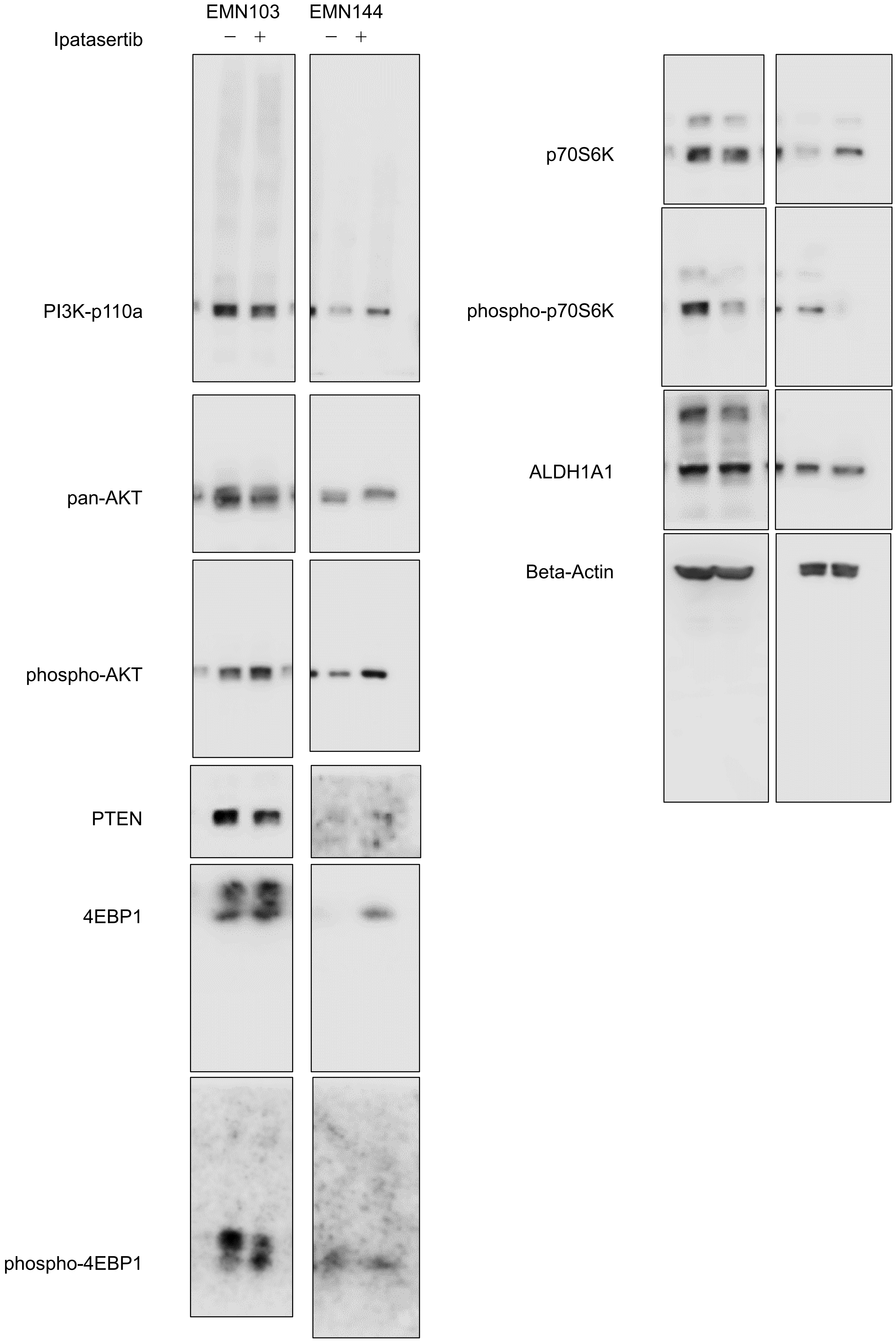

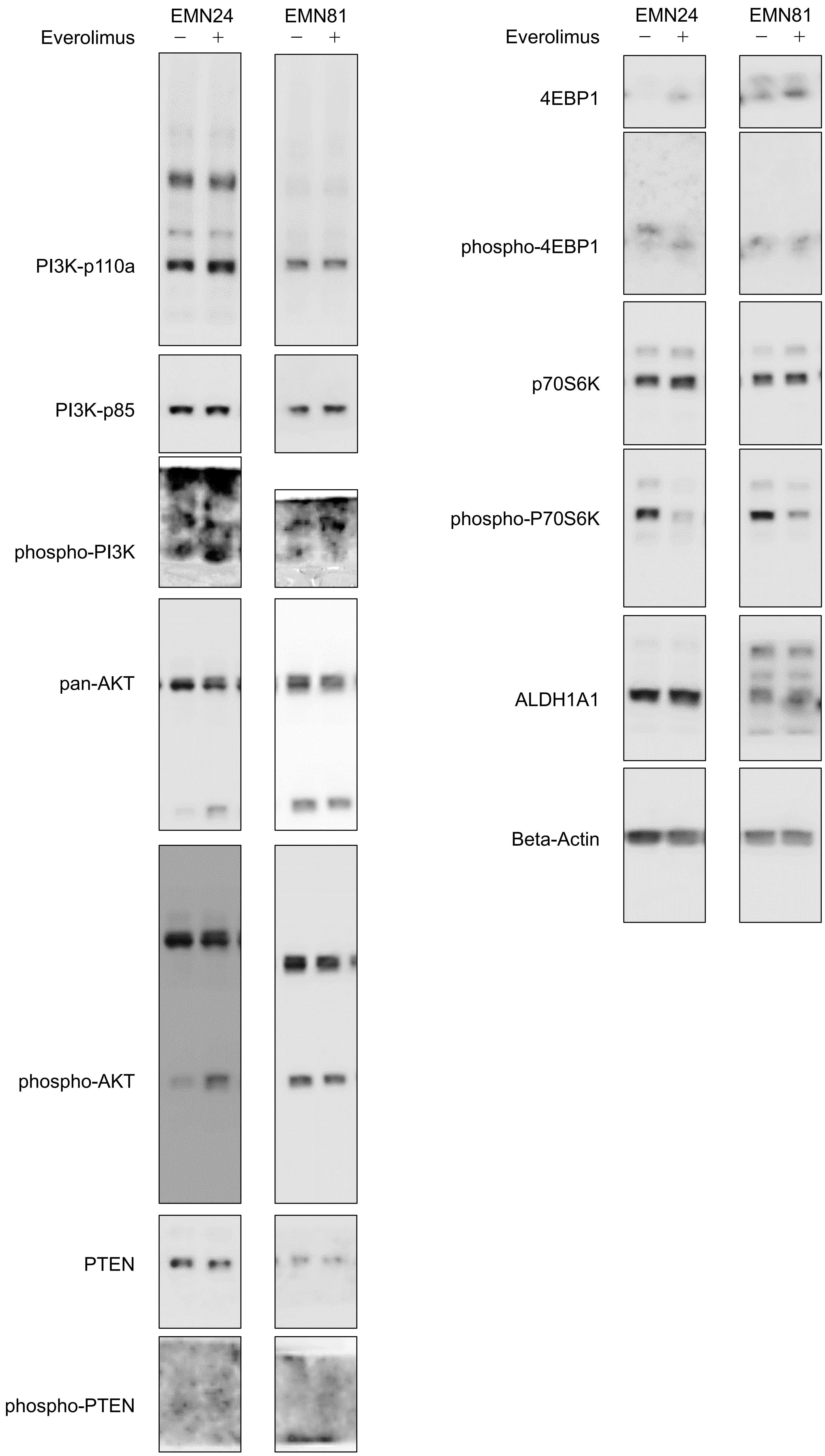
EMN24
EMN81
Everolimus
−
+
PI3K-p110a
PI3K-p85
phospho-PI3K
pan-AKT
phospho-AKT
PTEN
phospho-PTEN
4EBP1
phospho-4EBP1
p70S6K
phospho-P70S6K
ALDH1A1
Beta-Actin

Original Data Figure 4C



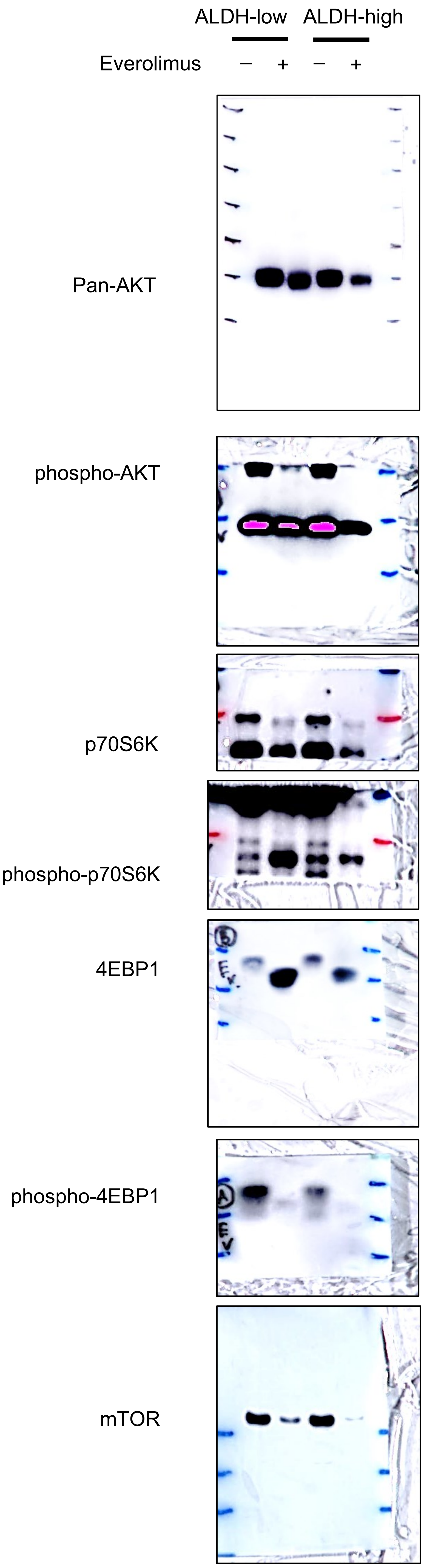


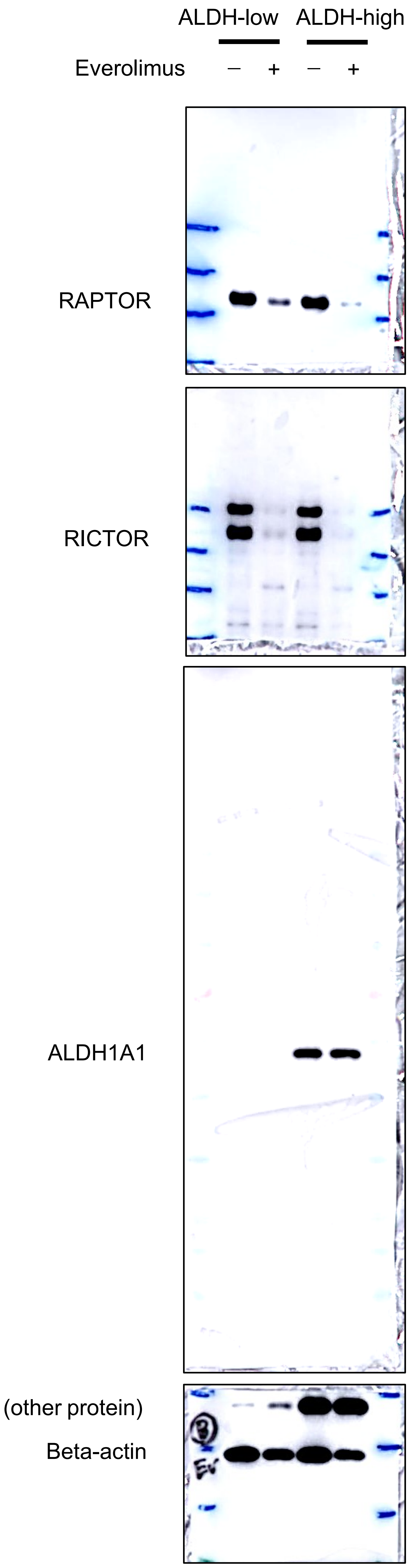

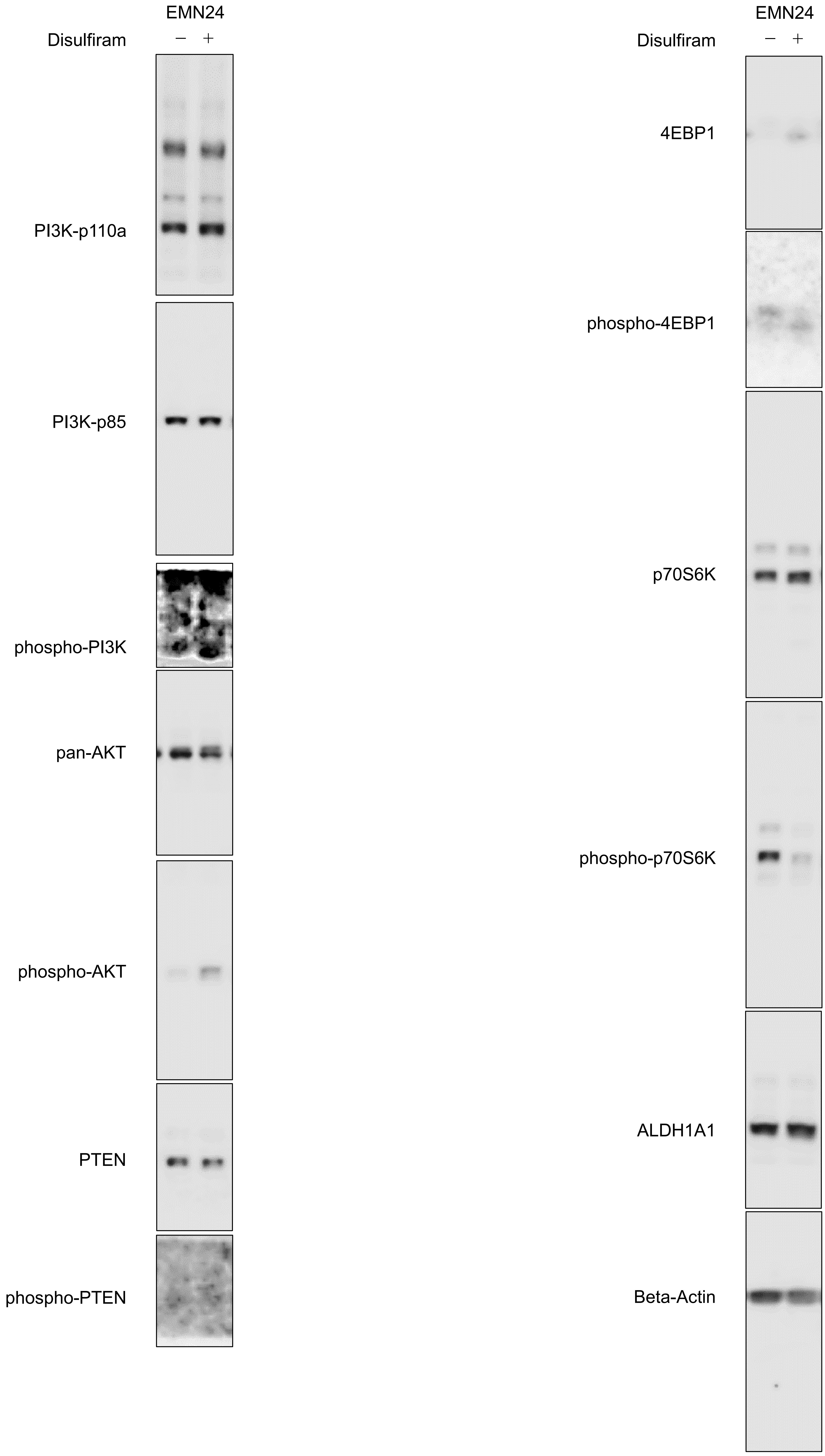
EMN24
Disulfiram
−
+
PI3K-p110a
PI3K-p85
phospho-PI3K
pan-AKT
phospho-AKT
PTEN
phospho-PTEN
EMN24
Disulfiram
−
+
4EBP1
phospho-4EBP1
p70S6K
phospho-p70S6K
ALDH1A1
Beta-Actin

ALDH-low ALDH-high

ALDH1A1

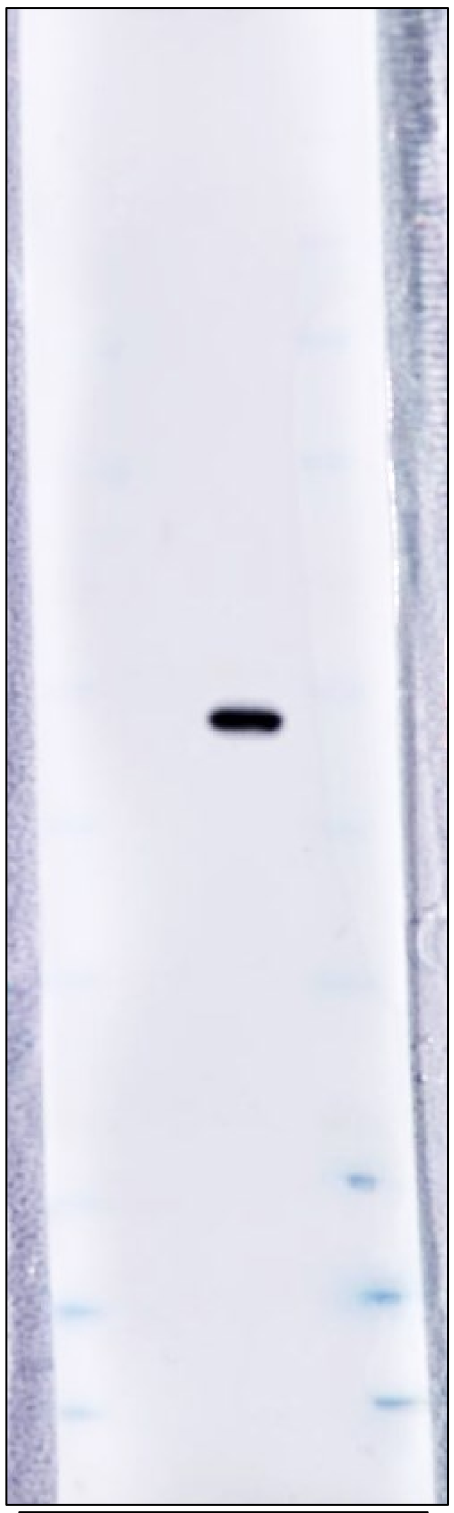

p70S6K

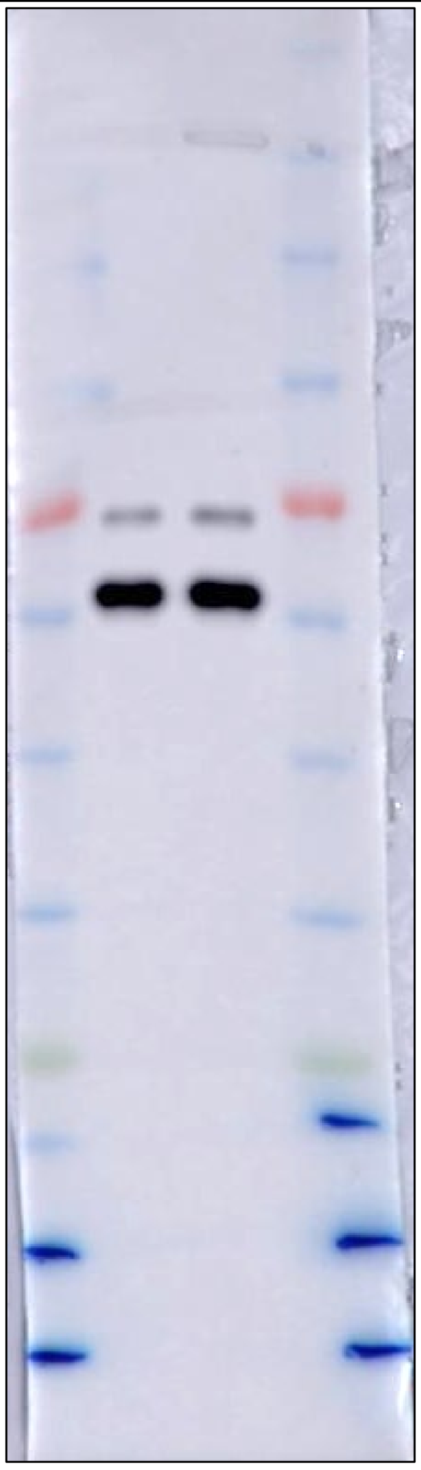

ALDH-low ALDH-high

phospho-p70S6K

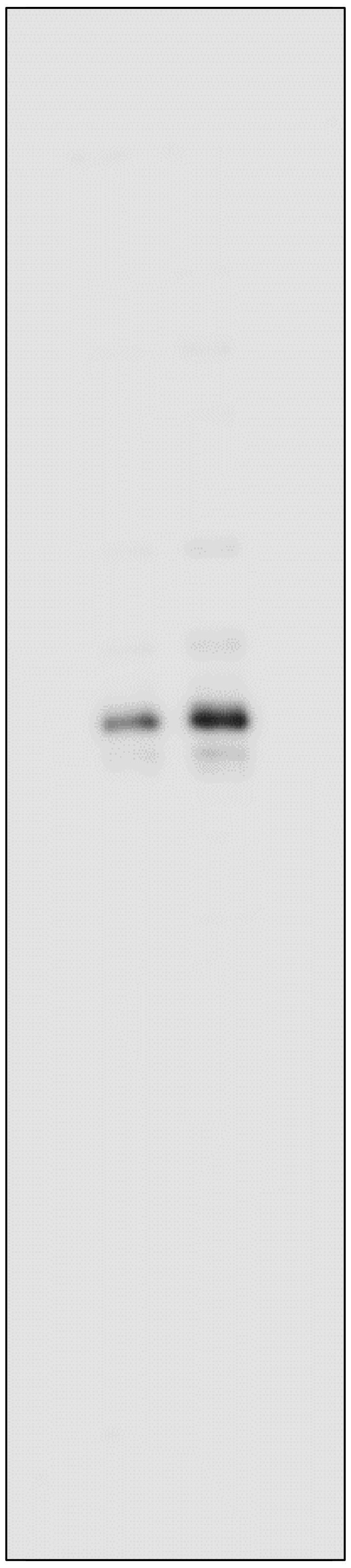

Beta-Actin

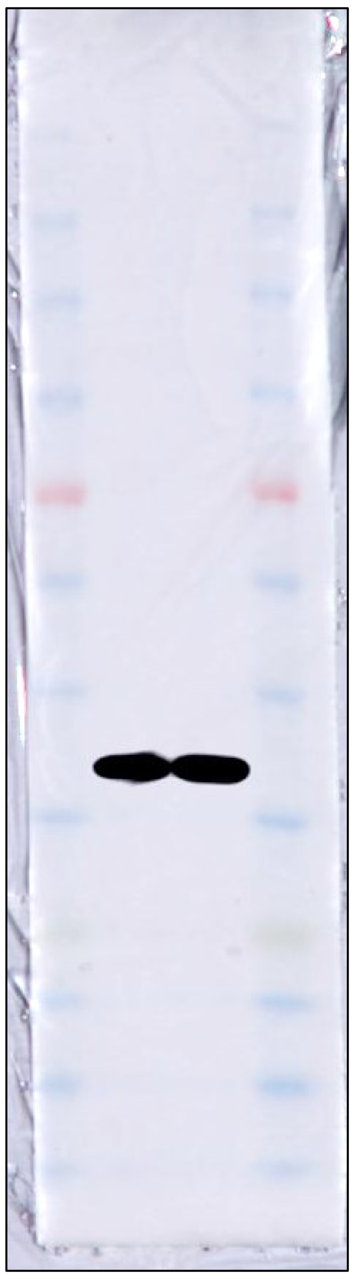

# Original Data Figure 5D



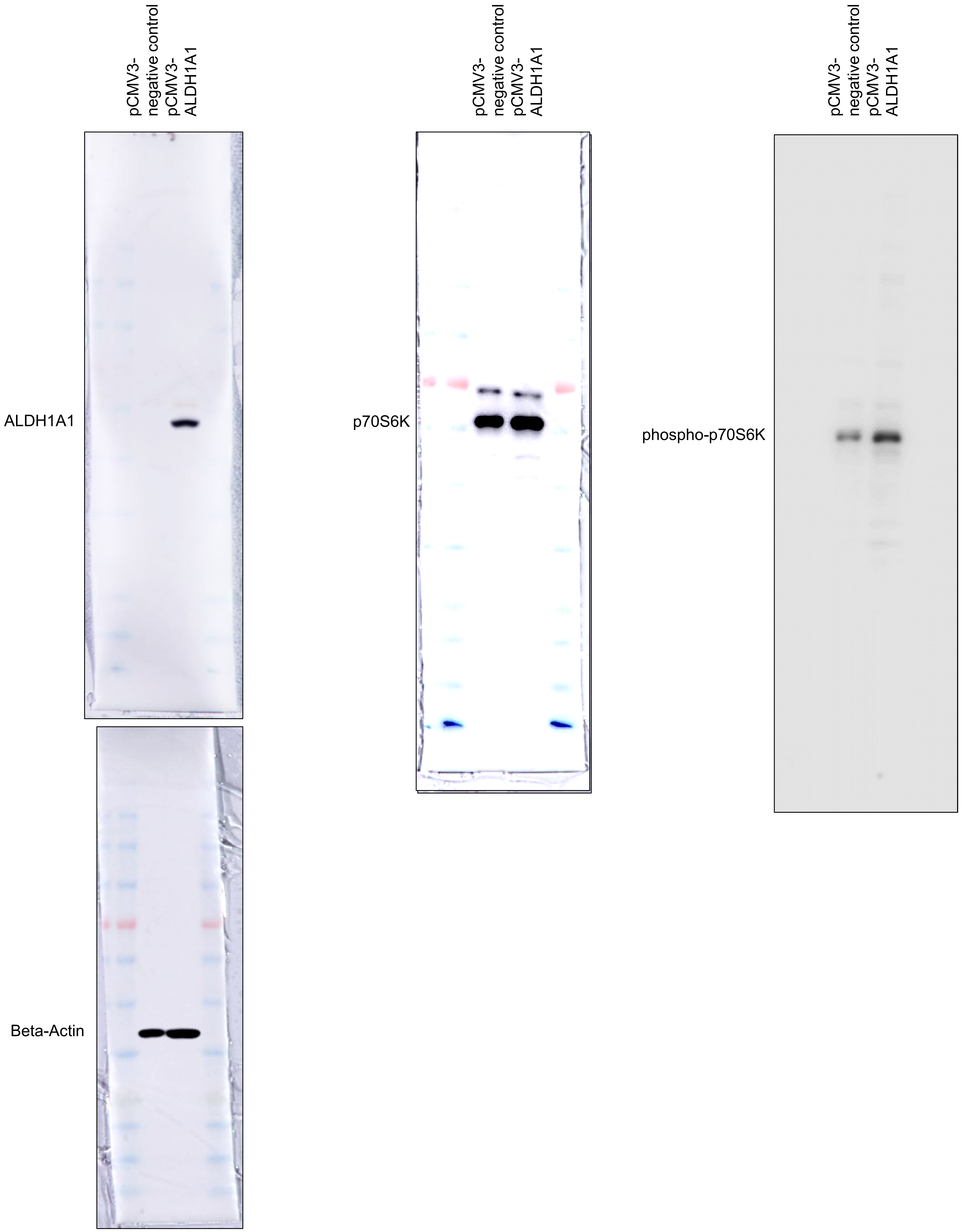

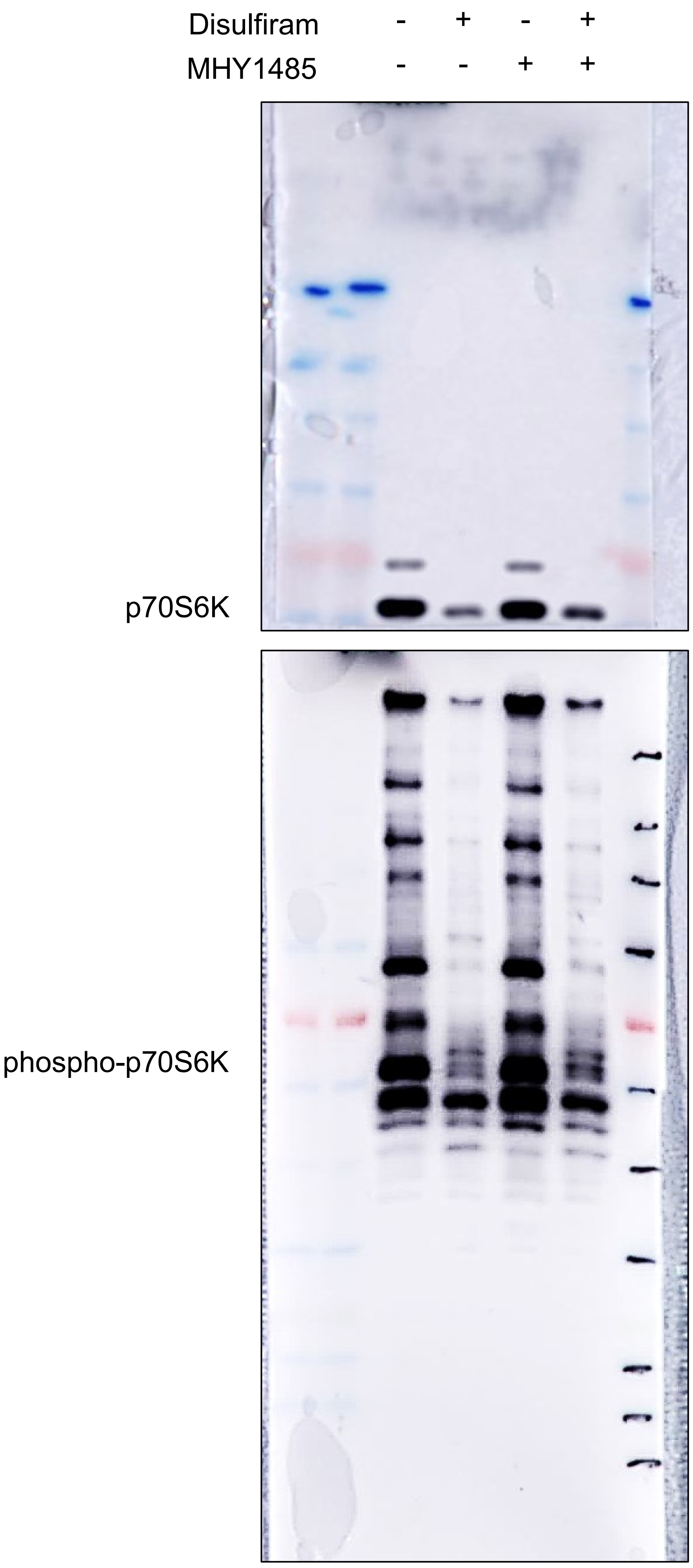
Disulfiram - + - +
MHY1485 - - + +
p70S6K
phospho-p70S6K

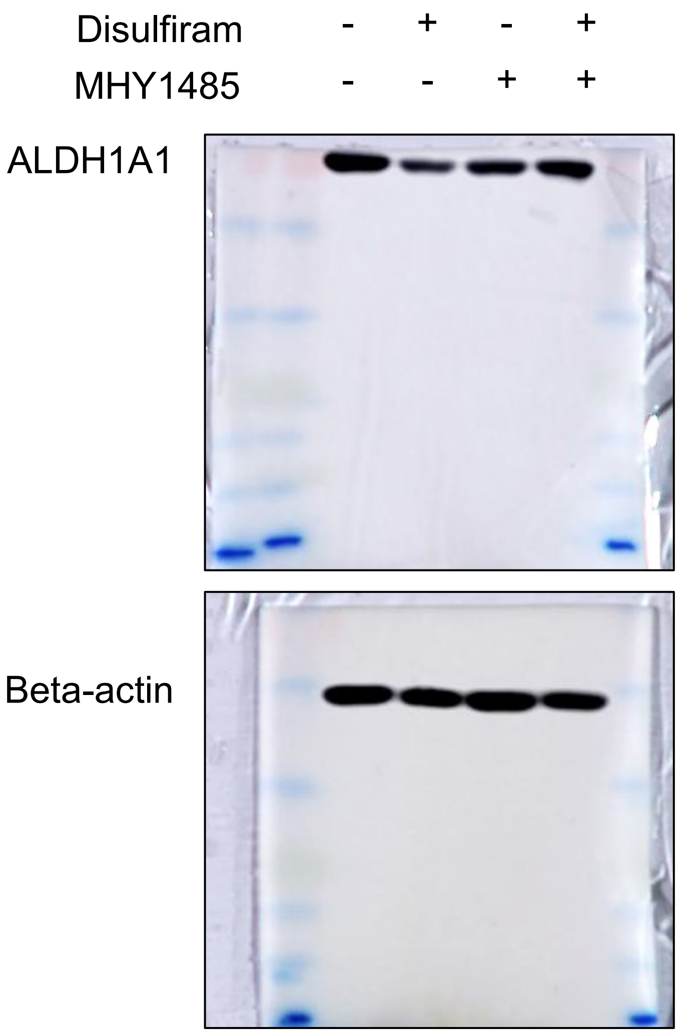
Disulfiram - + - +
MHY1485 - - + +
ALDH1A1
Beta-actin

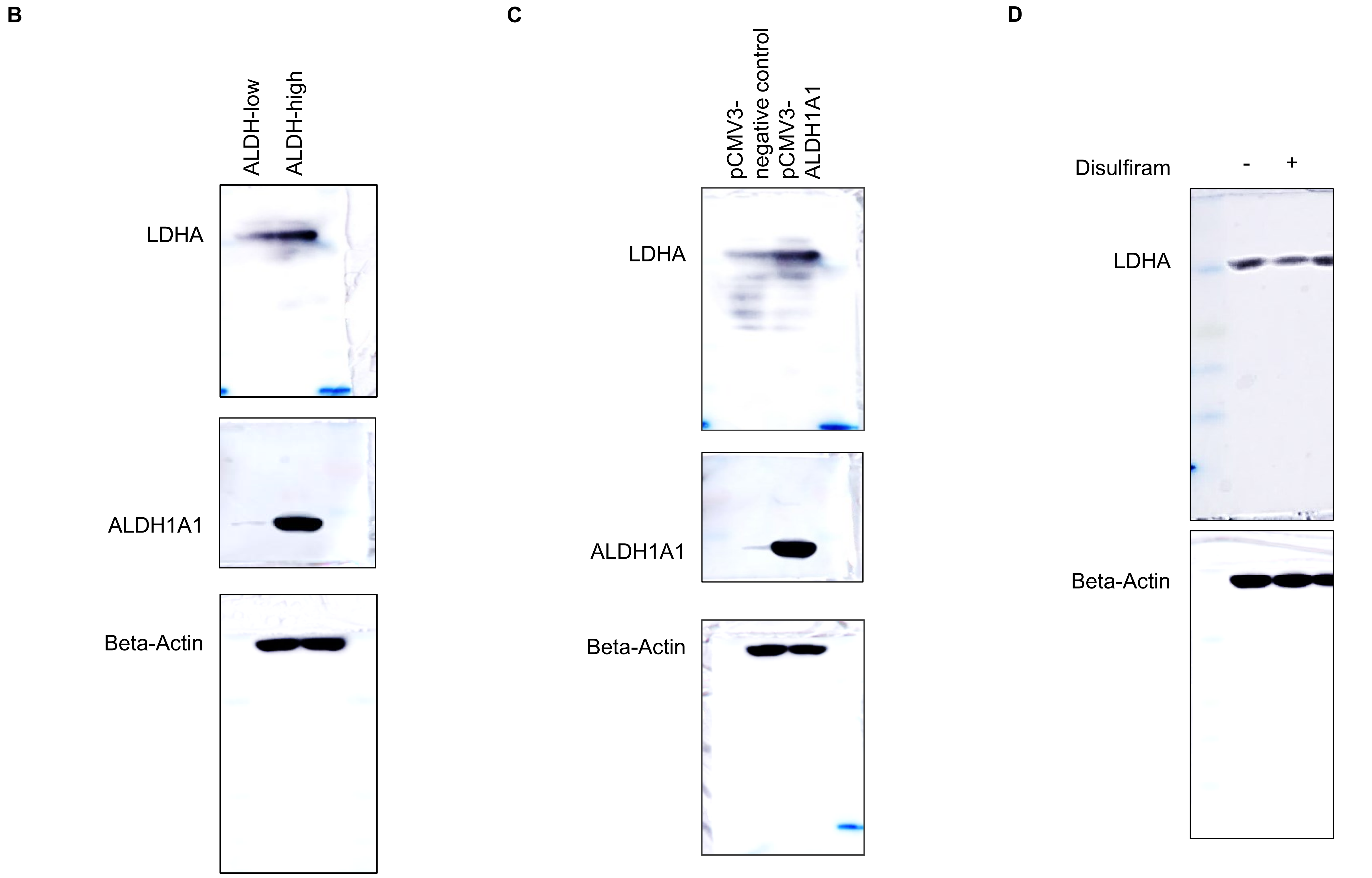
B
ALDH-low
ALDH-high
LDHA
ALDH1A1
Beta-Actin
C
pCMV3-
negative control
pCMV3-
ALDH1A1
LDHA
ALDH1A1
Beta-Actin
D
Disulfiram
-
+
LDHA
Beta-Actin

Original Data Figure 6H



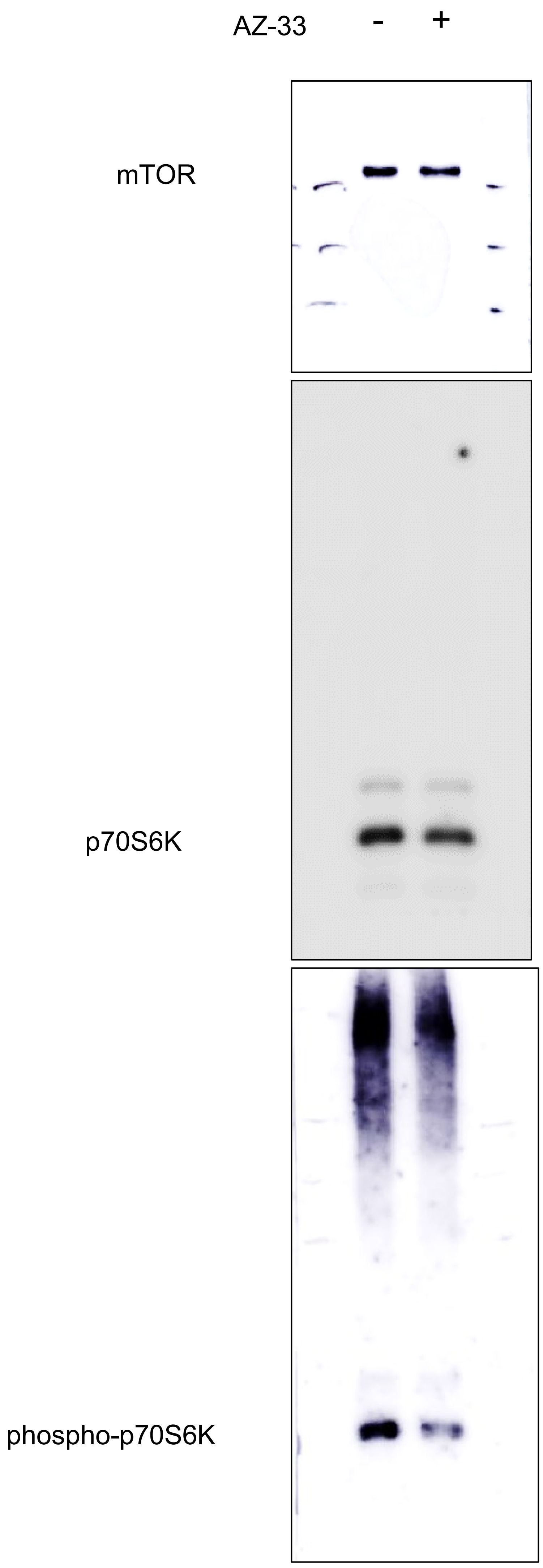


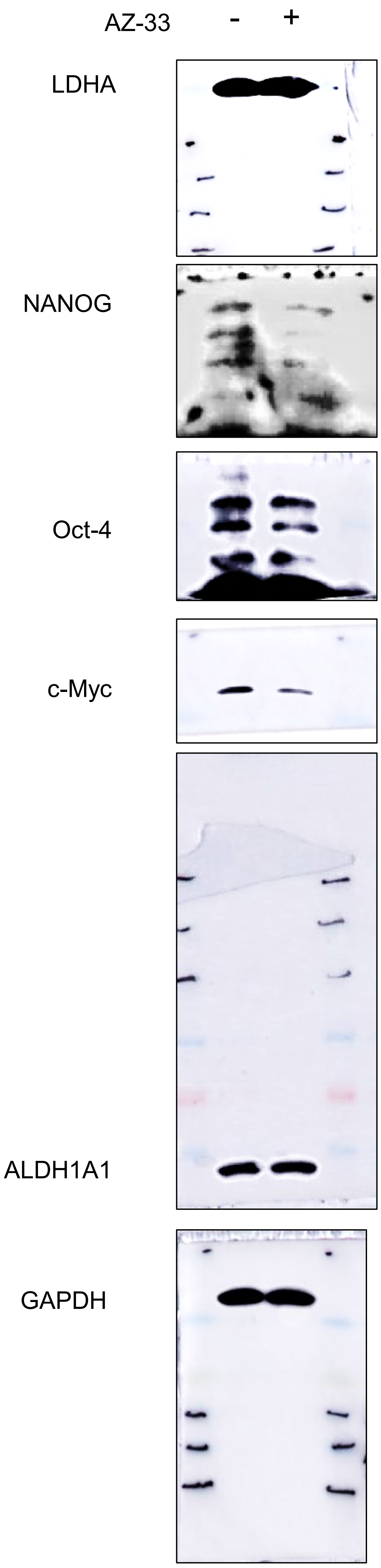

Original Data Figure 6J

Ueda *et al*

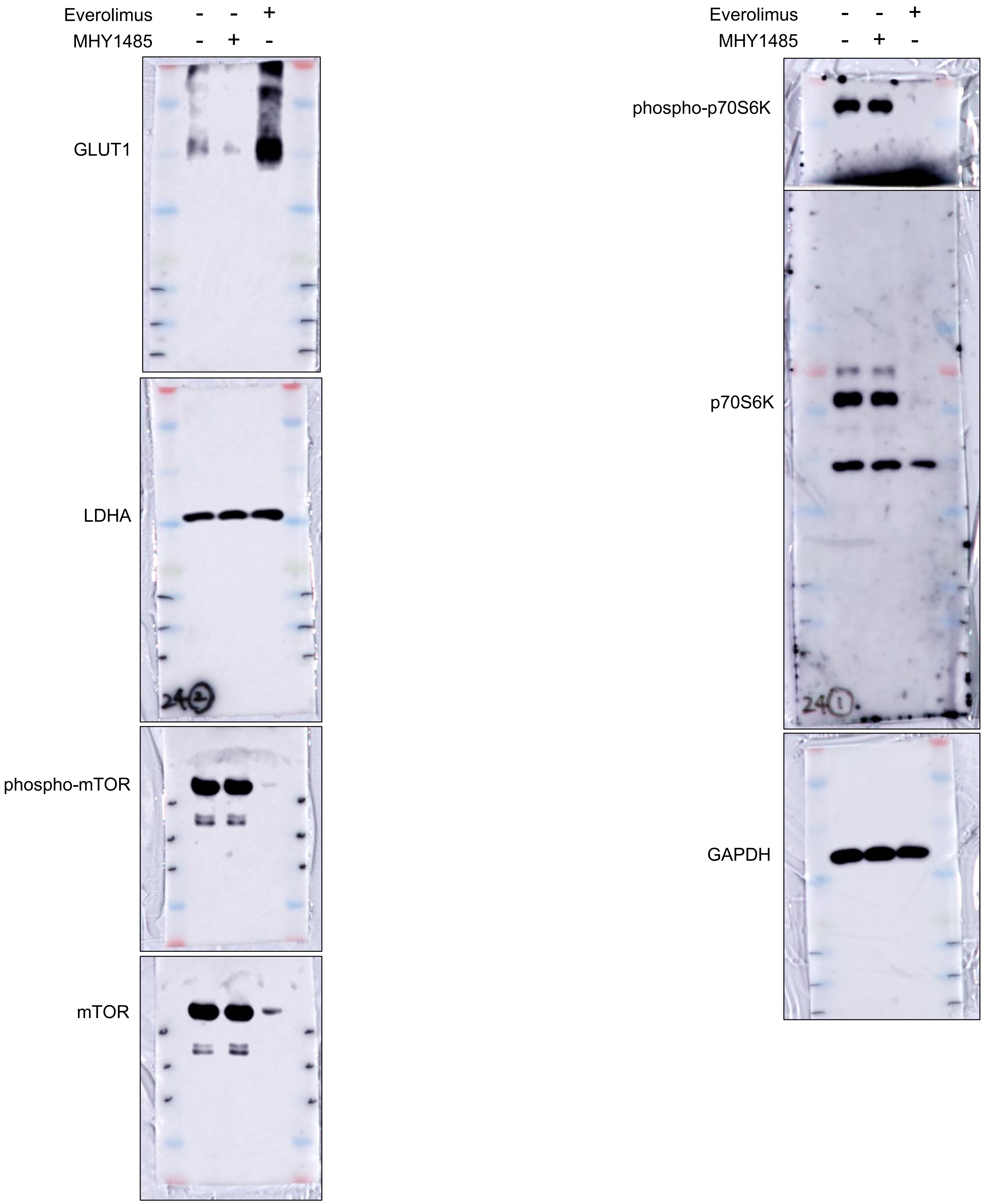

Ueda *et al*

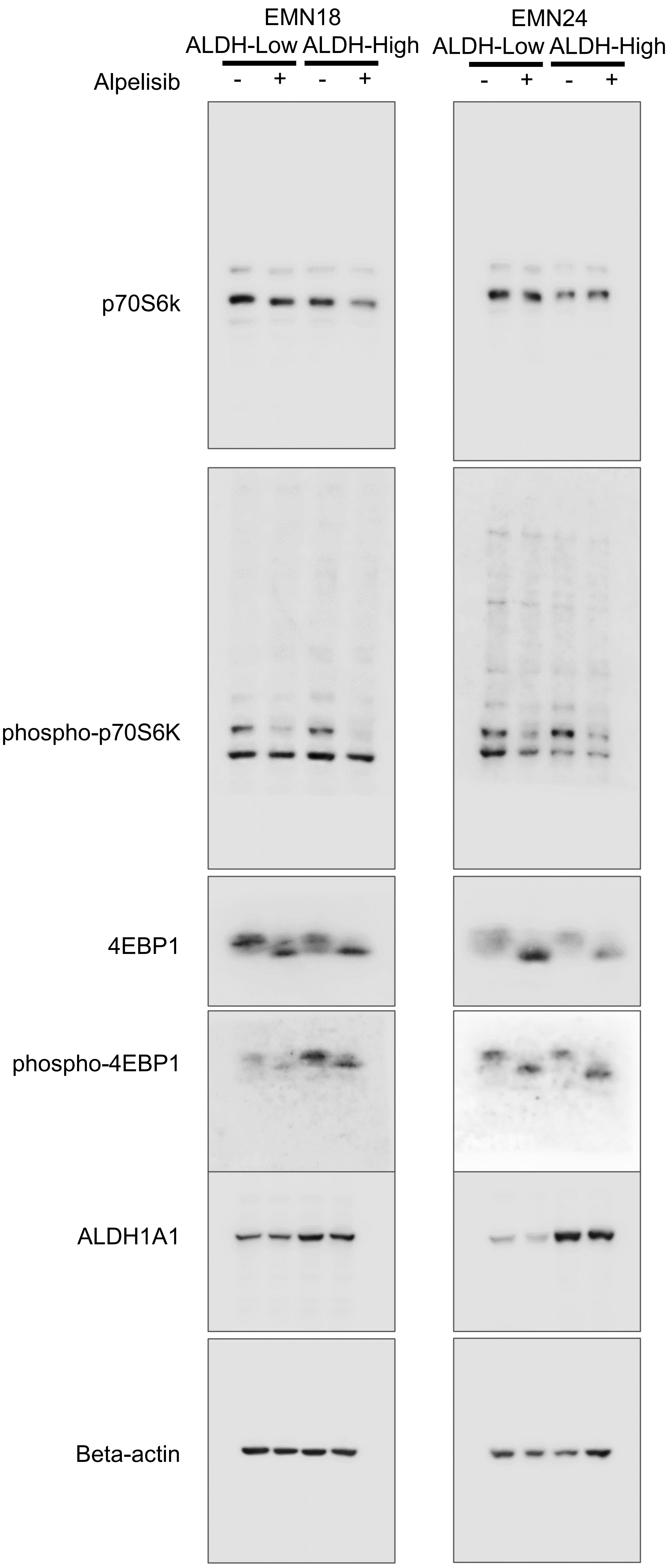
EMN18
ALDH-Low ALDH-High
EMN24
ALDH-Low ALDH-High
Alpelisib
- + - +
- + - +
p70S6k
phospho-p70S6K
4EBP1
phospho-4EBP1
ALDH1A1
Beta-actin

 

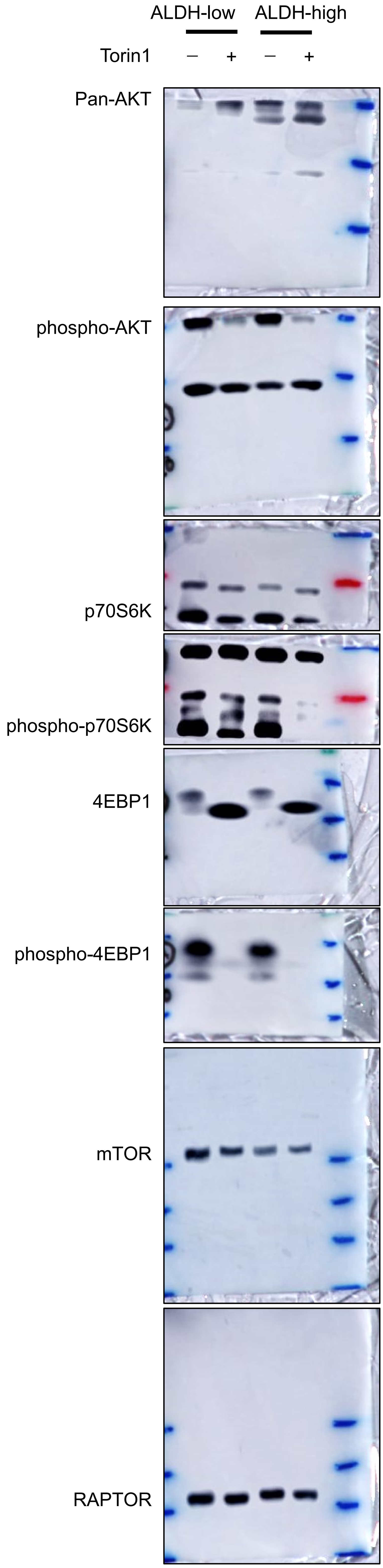
ALDH-low
ALDH-high
Torin1
–
+
–
+
Pan-AKT
phospho-AKT
p70S6K
phospho-p70S6K
4EBP1
phospho-4EBP1
mTOR
RAPTOR

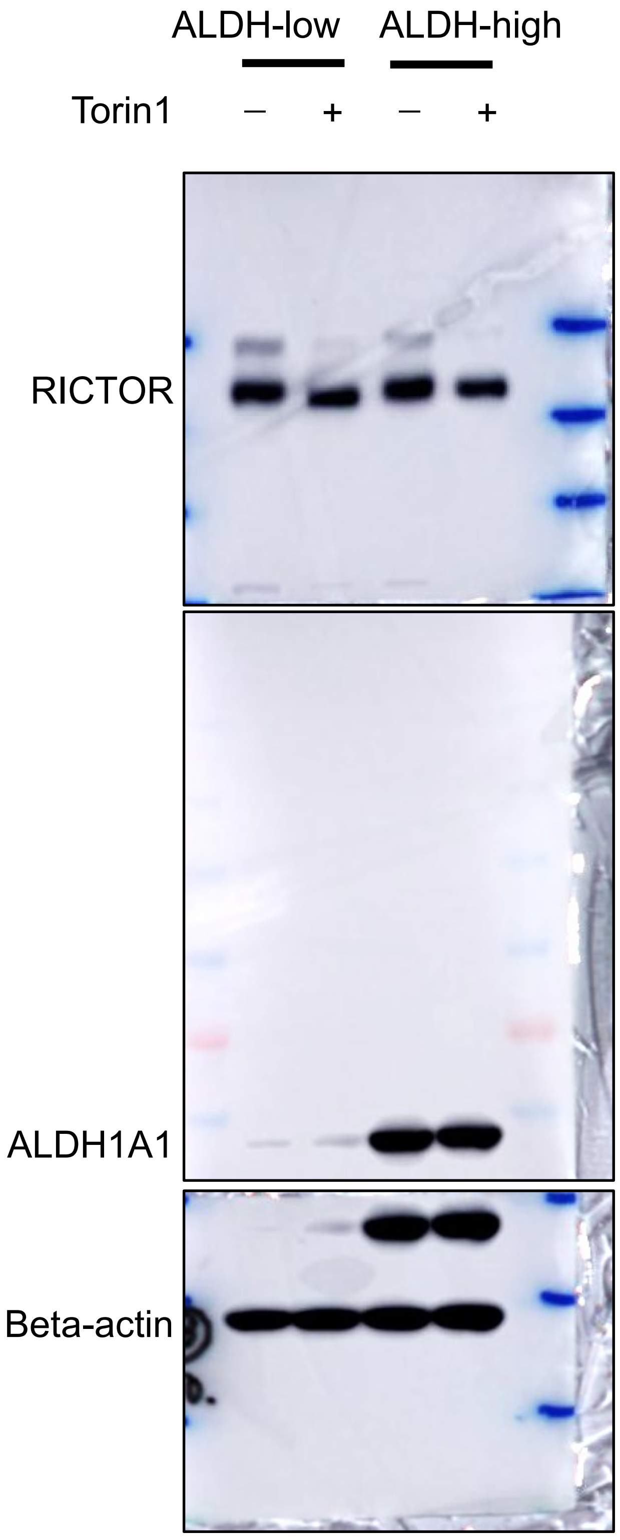
ALDH-low
ALDH-high
Torin1
–
+
–
+
RICTOR
ALDH1A1
Beta-actin

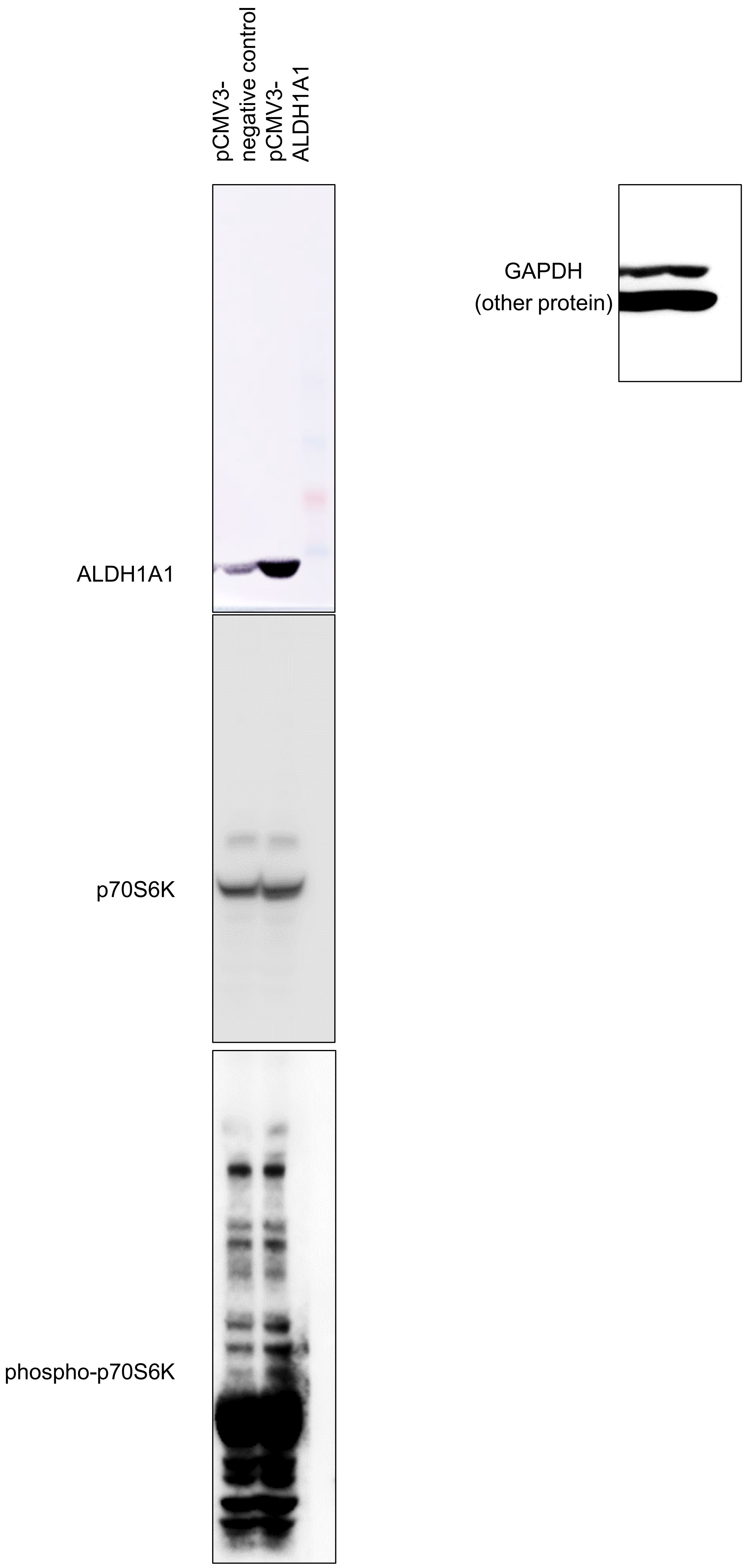
pCMV3-
negative control
pCMV3-
ALDH1A1
ALDH1A1
p70S6K
phospho-p70S6K
GAPDH
(other protein)

Original Data Figure S8A

Ueda *et al*

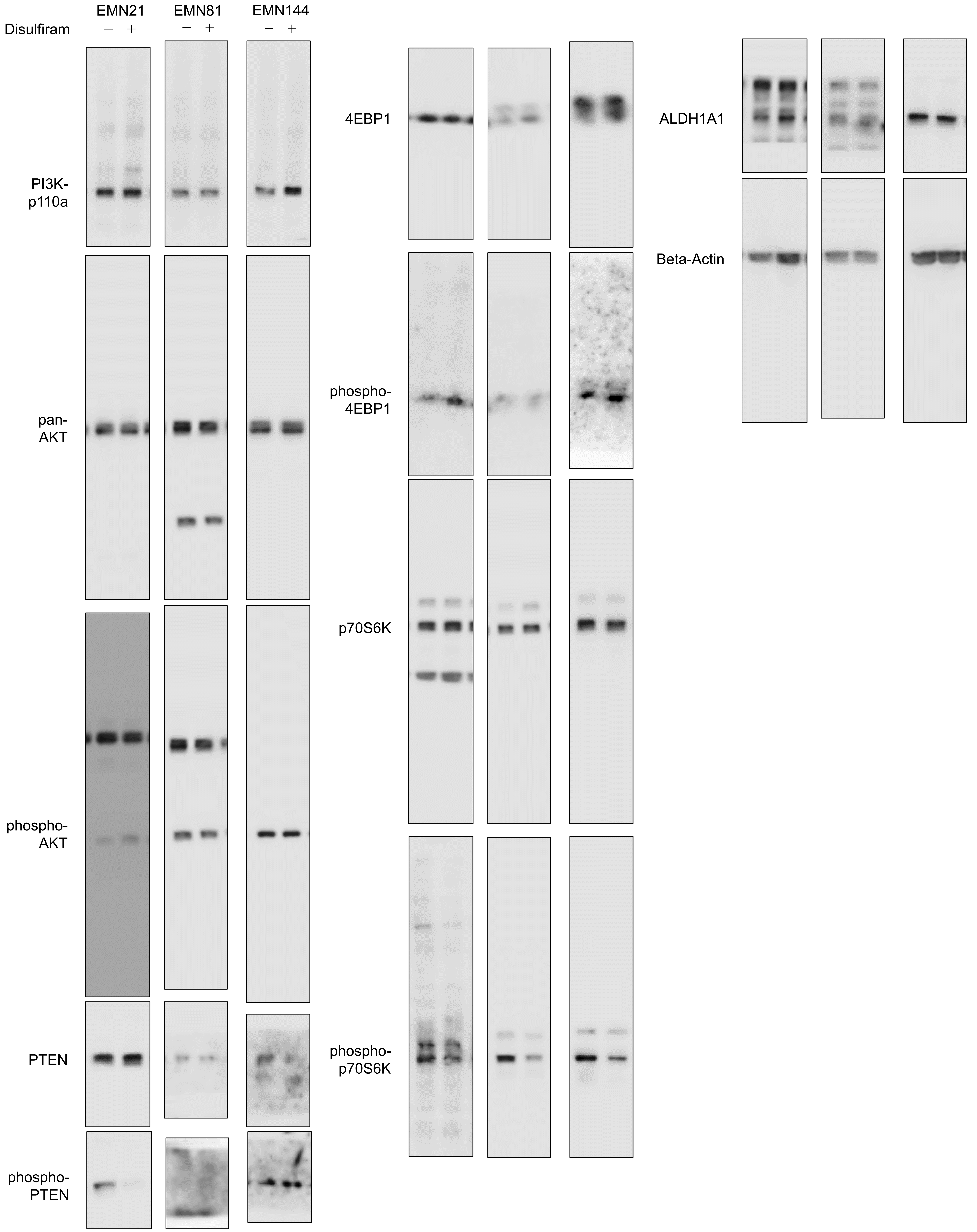

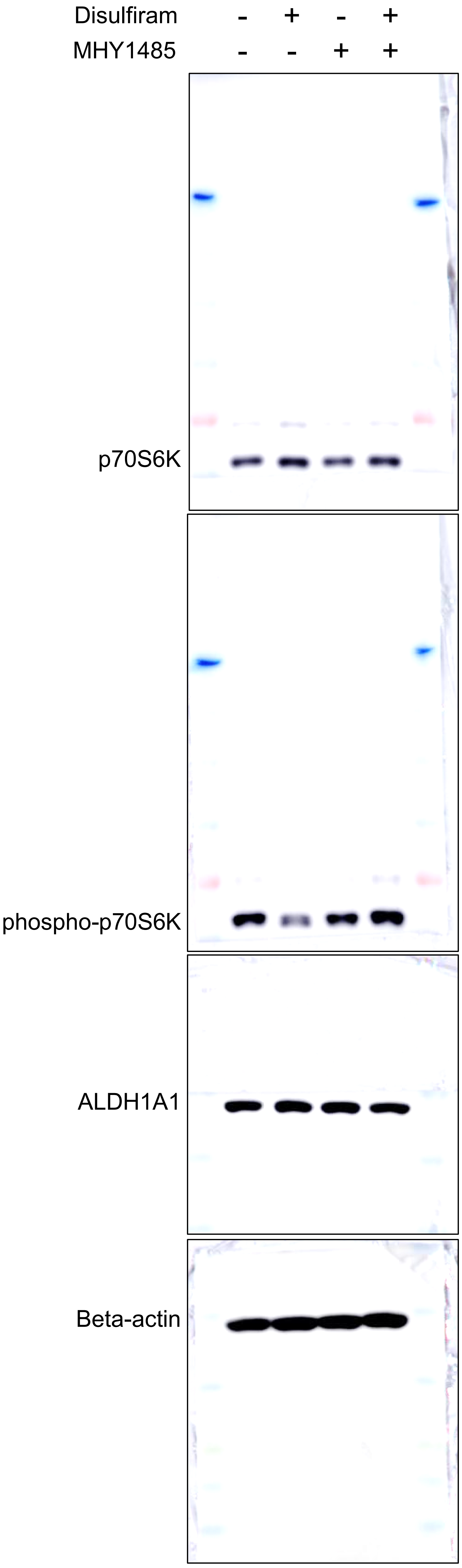
Disulfiram - + - +
MHY1485 - - + +
p70S6K
phospho-p70S6K
ALDH1A1
Beta-actin

**G**

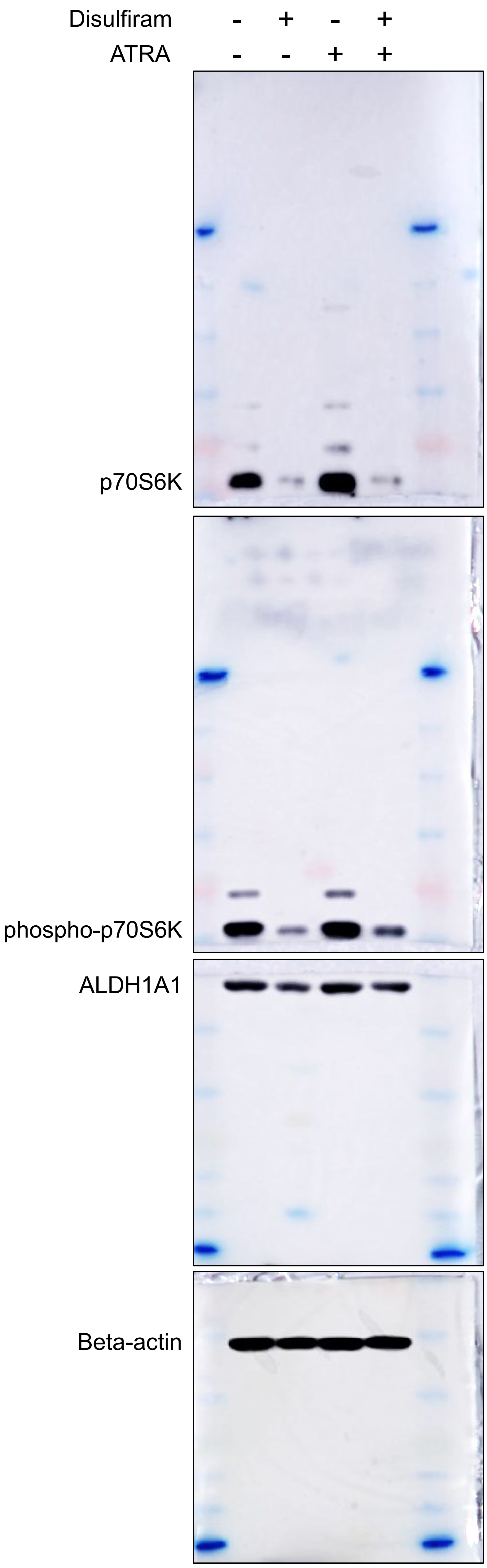
Disulfiram - + - +
ATRA - - + +
p70S6K
phospho-p70S6K
ALDH1A1
Beta-actin

**H**

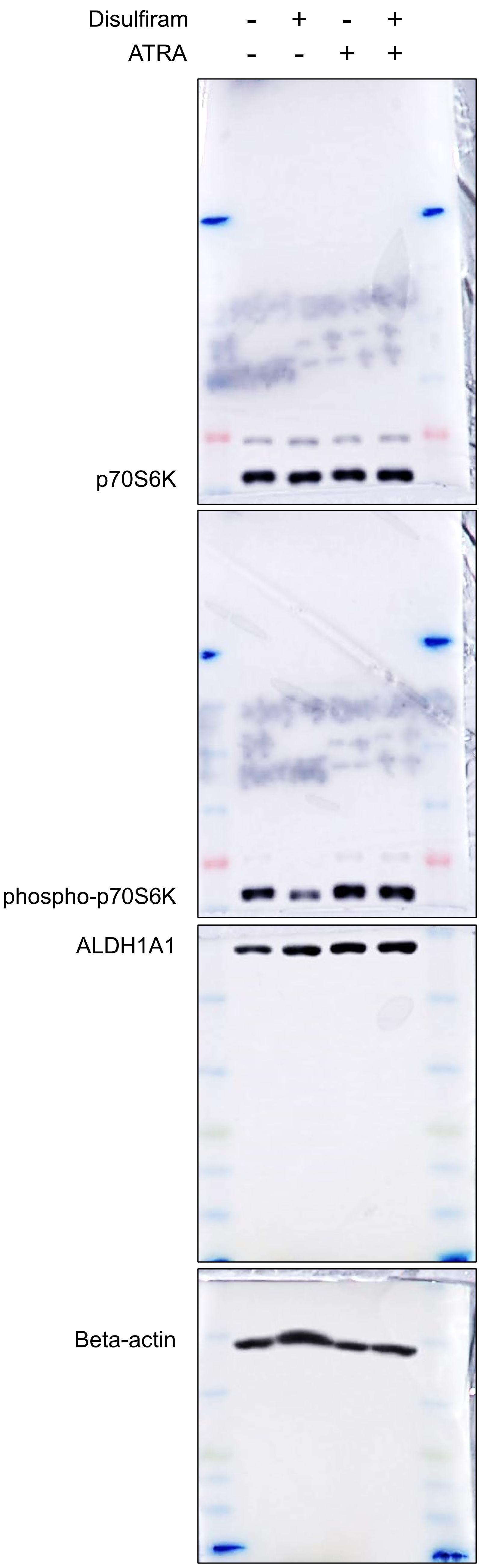
Disulfiram - + - +
ATRA - - + +
p70S6K
phospho-p70S6K
ALDH1A1
Beta-actin

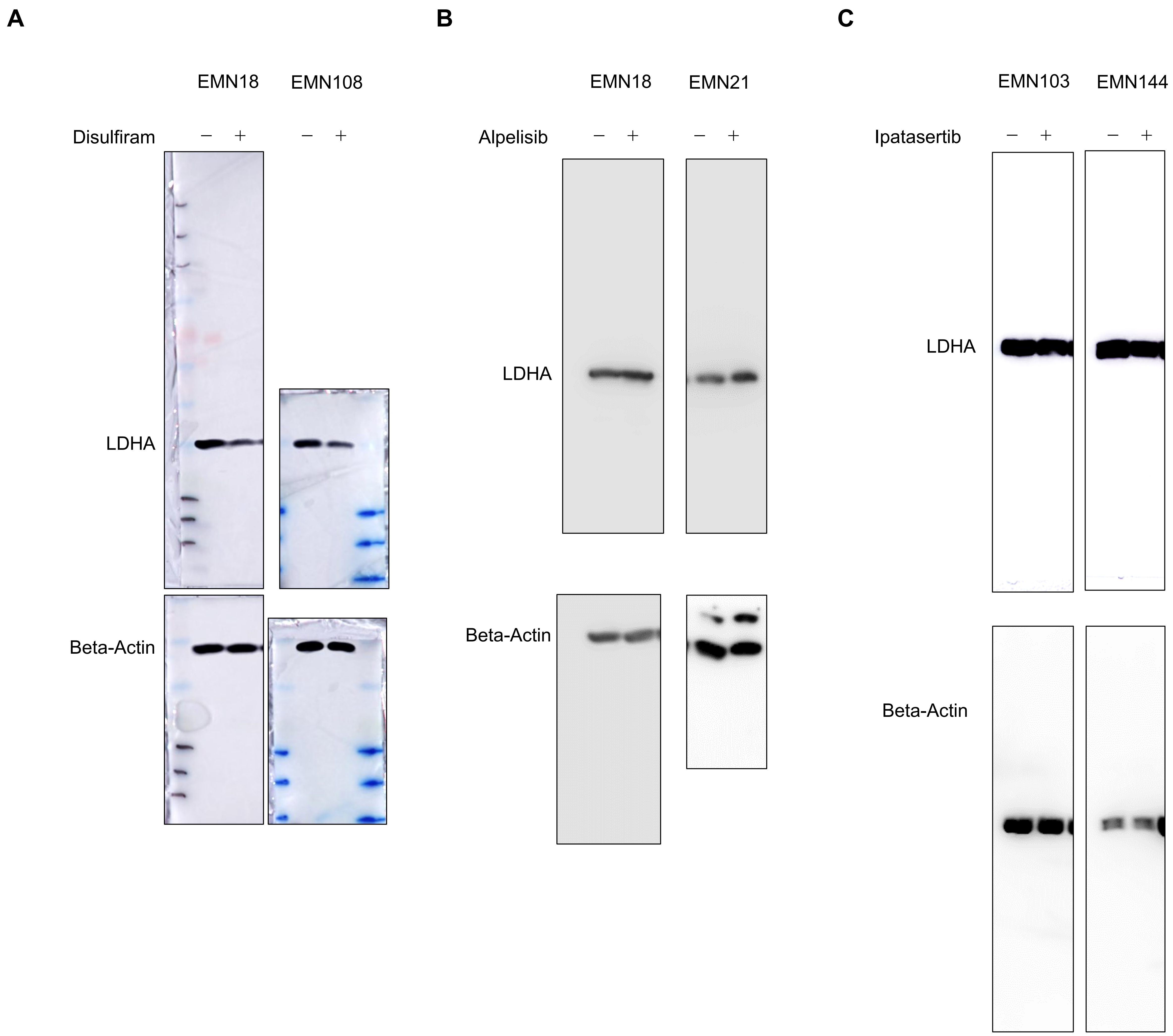
A
EMN18
EMN108
Disulfiram
–
+
–
+
LDHA
Beta-Actin
B
EMN18
EMN21
Alpelisib
–
+
–
+
LDHA
Beta-Actin
C
EMN103
EMN144
Ipatasertib
–
+
–
+
LDHA
Beta-Actin

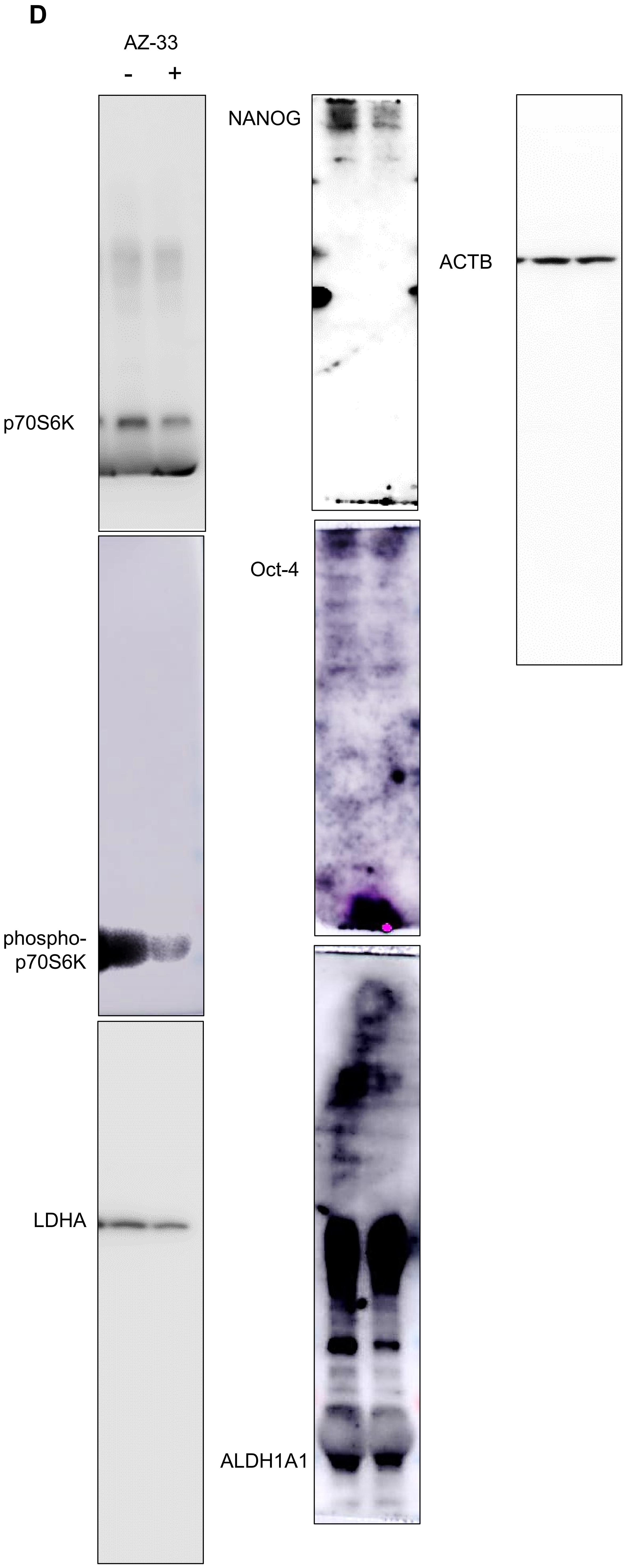
D
AZ-33
-
+
p70S6K
NANOG
ACTB
Oct-4
phospho-
p70S6K
LDHA
ALDH1A1

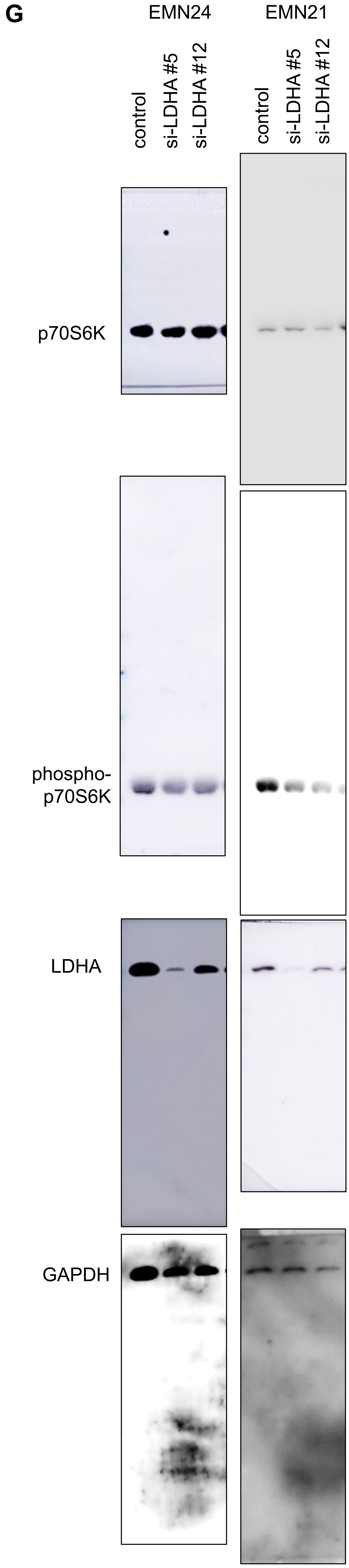
G
EMN24
EMN21
control
si-LDHA #5
si-LDHA #12
control
si-LDHA #5
si-LDHA #12
p70S6K
phospho-
p70S6K
LDHA
GAPDH

Original Data Figure S11A

Ueda *et al*

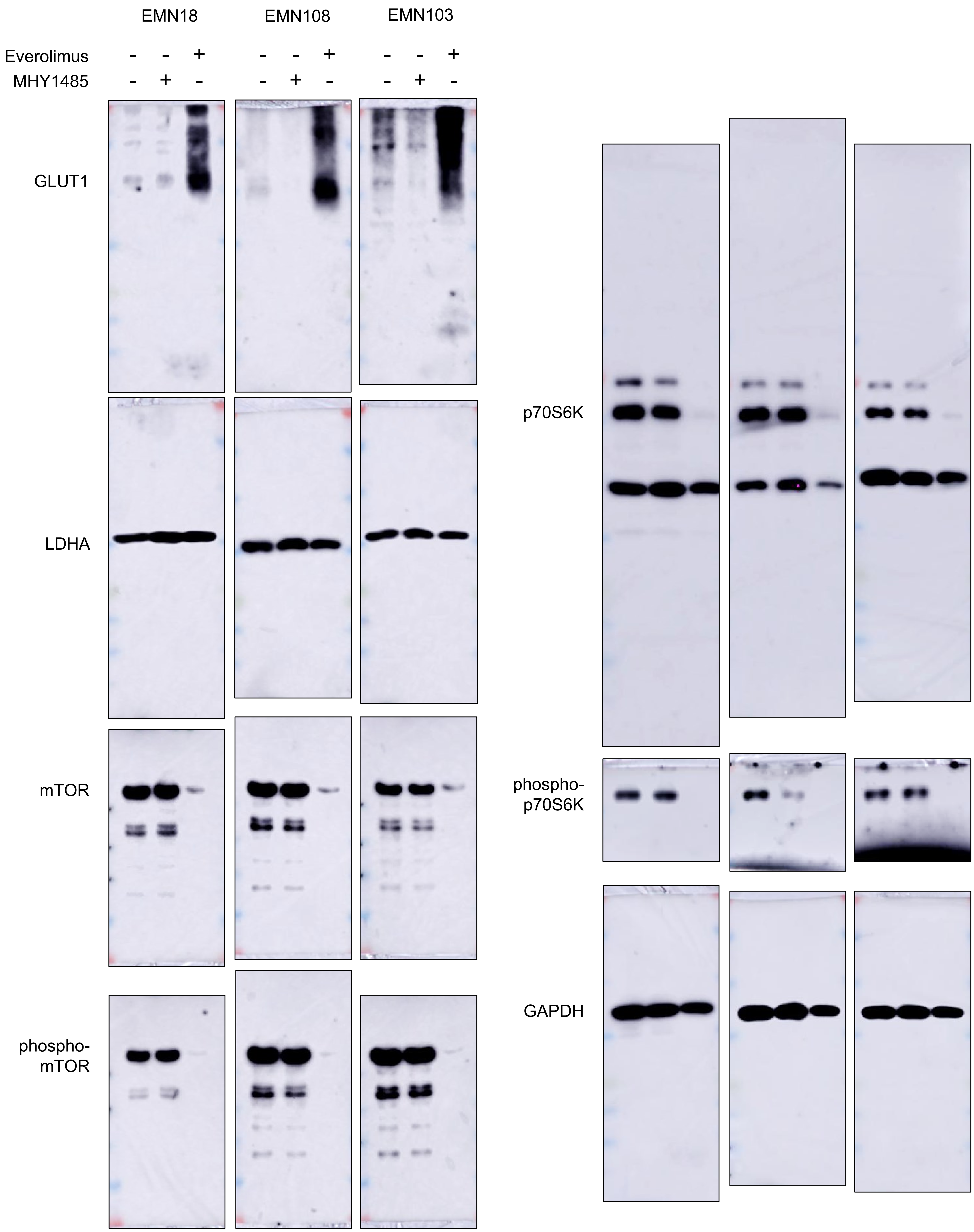

Supplement: Supplementary file 4 — original western blot figure [file 41420_2024_2204_MOESM4_ESM.pdf]
